# Simultaneous modification of *Thermotoga maritima* encapsulin subunits to produce multivalent nanoparticle vaccines for non‐typhoidal *Salmonella enterica*

**DOI:** 10.1111/febs.70561

**Published:** 2026-04-28

**Authors:** Carly A. Charron, Angelo Kaldis, Shabnam Shamriz, Moussa S. Diarra, Christopher P. Garnham, Rima Menassa

**Affiliations:** ^1^ Biology Department Western University London Canada; ^2^ London Research and Development Centre, Agriculture and Agri‐Food Canada London Canada; ^3^ Guelph Research and Development Centre, Agriculture and Agri‐Food Canada Guelph Canada; ^4^ Biochemistry Department Western University London Canada; ^5^ Present address: Guelph Research and Development Centre, Agriculture and Agri‐Food Canada Guelph Canada

**Keywords:** multivalent subunit vaccine, plant molecular farming, protein nanoparticle engineering, *Salmonella*, *Thermotoga maritima* encapsulin

## Abstract

Protein nanoparticles are increasingly used in medical applications such as drug delivery and antigen display. To fully harness their potential, it is essential to assess their capacity for surface modification and optimize their stability and production. In this study, we investigated the immunogenicity of *Thermotoga maritima* encapsulin (TmENC) displaying a 28 amino acid peptide from FepA, a *Salmonella* outer membrane protein, which we had previously characterized. In BALB/c mice, immunization induced a specific antibody response against the surface‐displayed epitope. We also evaluated the modularity of TmENC by introducing individual modifications at four distinct sites within the protein subunit, as well as simultaneous modification of up to four sites per subunit. All TmENC variants were transiently expressed in *Nicotiana benthamiana*, where they successfully assembled into nanoparticles; however, their accumulation and stability varied depending on the number and type of modifications introduced. These results demonstrate the versatility of TmENC as an antigen display platform and underscore its potential for developing multivalent vaccines through genetic modification of multiple sites within a single protein subunit.

## Graphical abstract

This study investigates the modularity of encapsulin—a protein nanoparticle—by simultaneously inserting up to four distinct *Salmonella* epitopes within the encapsulin subunit. The encapsulin variants were transiently expressed in plants where they assembled into nanoparticles; however, their accumulation and stability varied depending on the characteristics of the inserted epitopes. This study demonstrates the potential of encapsulin for antigen display, highlighting its capacity for the development of multivalent vaccines.

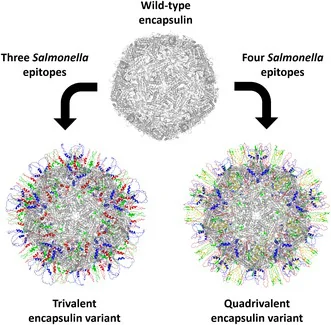

**Abbreviations**
C1: CirA single epitope TmENC variant
C2: CirA two‐epitope TmENC variant
C3: CirA three‐epitope TmENC variant
CHL: Chloroplast
CirA: Colicin receptor A
CYT: Cytosol
DPI: Days post‐infiltration
ELISA: Enzyme‐linked immunosorbent assay
ENC‐P2‐VP8*: Encapsulin with C‐terminal genetic fusion of core region of rotavirus VP8 protein
ER: Endoplasmic reticulum
F3: FepA three‐epitope TmENC variant
F4: FepA four‐epitope TmENC variant
FepA: Ferric enterobactin receptor
FW: Fresh weight
HA: Hemagglutinin.
I3: IroN three‐epitope TmENC variant
IgA: Immunoglobulin A
IgG: Immunoglobulin G
IMAC: Immobilized metal affinity chromatography
IroN: Salmochelin‐iron receptor
L10: Loop 10
L11: Loop 11
L2: Loop 2
L2‐#: L2 TmENC variant with insertion of L2 at the respective amino acid position
L2X3: L2 TmENC variant with insertion of L2 at three distinct amino acid positions
L5: Loop 5
L7: Loop 7
L8: Loop 8
MW: Molecular weight marker
PBS: Phosphate‐buffered saline
pCLGG‐X: pCaMLite‐GoldenGate‐X
PNPs: Protein nanoparticles
RBC‐S‐TP: Chloroplast transit peptide from the small subunit of RuBisCo
SDS/PAGE: Sodium dodecyl‐sulfate polyacrylamide gel electrophoresis
SEC: Size‐exclusion chromatography
tCUP: Tobacco cryptic upstream promoter translational enhancer
TmENC: *Thermotoga maritima* encapsulin
UA: Uranyl acetate

## Introduction

In the past decade, protein nanoparticles (PNPs) have emerged as a biocompatible platform for various medical applications such as drug delivery and antigen display. PNPs self‐assemble into stable, multi‐subunit structures, including hollow cage‐like structures, offering opportunities for encapsulation and controlled release of therapeutics. Their repetitive subunit organization enables the display of antigens in a high‐density array, effectively promoting immune recognition and enhanced immunological responses [1, 2]. Several naturally occurring PNPs of eukaryotic and prokaryotic origin have been investigated for use in vaccine development [3, 4, 5, 6].

Encapsulins are a widely distributed and functionally diverse class of PNPs found inside many prokaryotes [7]. They self‐assemble from identical protein subunits into capsid‐like particles ranging from 20 to 50 nm in diameter, with icosahedral symmetries of either *T* = 1, *T* = 3, or *T* = 4 [7, 8, 9]. A unique feature of encapsulins is that they naturally function as nanocompartments, selectively encasing native cargo proteins, such as enzymes, via short C‐terminal encapsulation tags. This cargo‐loading capacity has been applied to therapeutic proteins [10, 11]. Additionally, the surface of encapsulin can be modified genetically, enabling the controlled insertion of a desired peptide within a specific position of the nanoparticle, resulting in repetitive display on the nanoparticle surface [12, 13, 14].

The encapsulin derived from the thermophilic bacterium *Thermotoga maritima* (TmENC: 60mer, 20–24 nm, *T* = 1) is the most extensively studied [15]. The TmENC subunit consists of three domains, P, A, and E, and contains ten sites which have been genetically modified [12, 14, 16, 17, 18, 19, 20]. Of these ten sites, eight are surface exposed: site K239‐D240 within the P domain; sites E124‐K125, E135‐E136, K138‐I139, and the C terminus (F265) within the A domain [12, 19, 20]; and sites E54‐V55, V57‐L58, E61‐N62, and G68‐L69 within the E domain [14, 18, 20]. Previous studies have suggested that the C terminus is less accessible [14]; however, its accessibility can be enhanced through the addition of a linker between TmENC and the added peptide [20]. Modification of sites E61‐N62, E124‐K125, E135‐E136, K138‐I139, and K239‐D240 has resulted in high purification yield of stable TmENC nanoparticles [19, 20], while modification of sites E54‐V55, V57‐L58, and G68‐L69 leads to unstable or insoluble nanoparticles with low purification yield [18].

We previously engineered a TmENC variant with six histidines at site K239‐D240 of the subunit, and five TmENC variants with different epitopes from ferric enterobactin receptor (FepA), a *Salmonella* outer membrane protein, inserted at site K138‐I139, in addition to six histidines inserted at site K239‐D240 [19]. For all variants, we achieved a high yield of assembled TmENC nanoparticles upon expression in *Nicotiana* spp., thus indicating that the TmENC subunit can tolerate peptide insertions at multiple modification sites.

FepA is a *Salmonella* outer membrane protein involved in iron acquisition. It has a large beta‐barrel structure that spans the outer membrane and contains large extracellular loops that facilitate iron uptake [21, 22]. The extracellular loops of FepA are attractive target antigens for vaccine development as they are surface‐exposed on the bacteria, where they would be visible to the immune system, and antibodies that recognize them may inhibit bacterial iron uptake and prevent *Salmonella* colonization within the host [23]. However, the immune response against a single epitope is generally not enough to protect against infection [24, 25, 26], and exposure to multiple FepA epitopes has the potential to generate a more protective immune response. Moreover, the outer membrane of *Salmonella* contains additional iron acquisition proteins, colicin receptor A (CirA) and salmochelin‐iron receptor (IroN), that are functionally redundant [27]. Thus, antibodies targeting FepA alone may not inhibit *Salmonella* colonization, as CirA and IroN may compensate for impaired FepA function. Therefore, producing multivalent TmENC nanoparticles targeting all three iron acquisition proteins may be a more promising route to provide protection. This prompted us to explore the extent to which the modification sites within the TmENC subunit could be utilized to produce multivalent TmENC nanoparticles displaying multiple epitopes as a potential *Salmonella* vaccine for poultry.

TmENC variants have predominantly been produced in *Escherichia coli* [12, 14, 28, 29]; however, in this study, we utilized *Nicotiana. benthamiana* as an expression host. Plants are a cost‐effective and readily scalable production platform [30, 31, 32] and facilitate proper folding and assembly of structurally complex proteins such as PNPs and virus‐like particles [33, 34, 35]. Plants are also a uniquely versatile production platform capable of supporting both prokaryotic and eukaryotic protein expression. Unlike bacterial systems, plants can perform a wide range of post‐translational modifications, which can be tailored through subcellular targeting [36, 37]. Moreover, plant‐based production offers a unique advantage for oral delivery. For livestock vaccines, such as those targeting *Salmonella*, incorporating antigen‐containing plant material directly into animal feed could enable efficient, large‐scale immunization with minimal cost to farmers [38, 39].

In the present study, we investigated the immunogenicity of TmENC displaying a 28 amino acid peptide from FepA, referred to as L2, at site K138‐I139 which we had previously characterized [19]. We investigated the amenability of TmENC sites E61‐N62, E124‐K125, and K239‐D240 to the insertion of L2, as well as modification of the C terminus of TmENC via the addition of a 10xHis‐tag. We also assessed the amenability of the TmENC subunit to simultaneous peptide insertion at multiple modification sites using epitopes from the *Salmonella* iron acquisition proteins FepA, CirA, and IroN. We showed that in all cases, TmENC nanoparticles assembled successfully. These findings demonstrate the potential of TmENC for antigen display, highlighting its capacity for the development of multivalent vaccines through genetic modification of multiple sites within the TmENC subunit.

## Results

We previously evaluated monovalent TmENC variants containing epitopes from FepA (E‐L2, E‐L3, E‐L4, E‐L8, and E‐L10) [19]. Of these, we selected E‐L2 for an immunogenicity study in mice as loop 2 is critical for the recognition and import of iron‐loaded siderophores [40], and E‐L2 was one of the highest accumulating proteins of those previously assessed [19]. Mice were immunized with crude plant extract from *N. benthamiana* containing ~ 10 μg of E‐L2. Plant crude extracts containing vaccine candidates for poultry pathogens have been shown to induce neutralizing antibodies in vaccinated mice and chickens [41]. Nanoparticles typically confer strong adjuvanting properties which can aid in the induction of an immune response; however, this can also lead to the production of antibodies against the nanoparticle and not the target antigen [3]. Because of this, mice in the control group were injected with crude plant extract containing ~10 μg of unmodified TmENC to assess whether an immune response is induced against the TmENC nanoparticle itself, or if the response is specific to the L2 epitope.

E‐L2‐specific IgG antibody responses from individual mice were measured by enzyme‐linked immunosorbent assay (ELISA) using serial dilutions of the pre‐immune and final bleed sera by coating the ELISA plates with purified E‐L2. In the final bleeds, both treatments induced an antibody response with an endpoint titer of 512 000‐times dilution (Fig. 1A). For both the TmENC control and E‐L2 treatments, there was a significant difference in relative absorbance between the pre‐immune serum and the final bleed (8000‐times dilution; *P*‐value = 0.00054, and *P*‐value = 0.0099, respectively) (Fig. 1B). Additionally, a significant difference was observed between the final bleeds from the control TmENC and E‐L2 treatments (*P*‐value = 0.0188), indicating that there is a significant immune response to FepA loop 2.

**Fig. 1:**
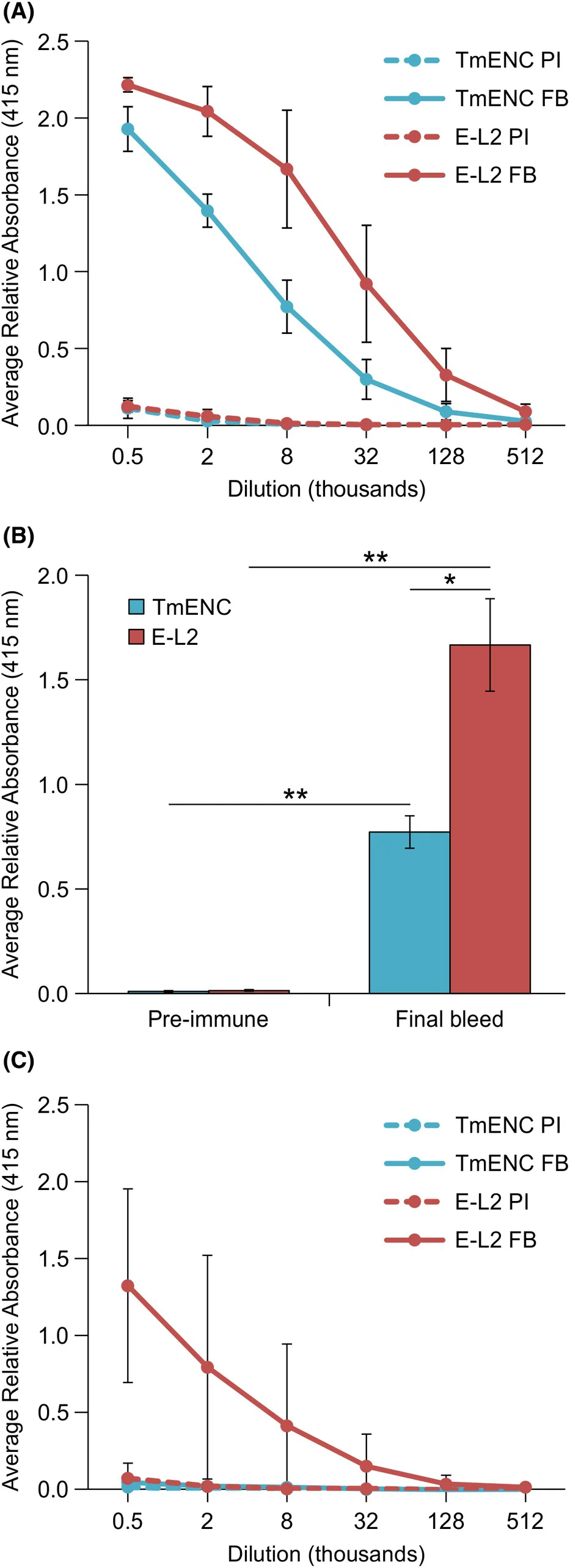
Immunization with E‐L2 induces an epitope‐specific antibody response in mice. Results are representative of two experiments where all sample dilutions were run in duplicate. Sample sizes of the control and experimental groups are *n* = 6 and *n* = 4, respectively. (A) Average enzyme‐linked immunosorbent assay (ELISA) absorbances across a dilution series of mouse sera for the TmENC control and E‐L2 experimental treatments. For both treatments, the ELISA plates were coated with purified E‐L2 to assess the antigen‐specific antibody response. Pre‐immune bleeds (PI) are represented by dotted lines and final bleeds (FB) by solid lines. Error bars represent the standard deviation of the mean. (B) Average antibody responses to E‐L2 for the TmENC control and E‐L2 experimental treatments detected using ELISA plates coated with purified E‐L2. All data shown is from the 8000‐times sera dilution. Absorbance values were averaged across biological replicates for each treatment group (± standard error of the mean) and significance (* = *P‐*value < 0.05; ** = *P*‐value < 0.01) was determined using a one‐tailed Student's *t*‐test for unequal variance followed by a Holm‐Bonferroni correction for multiple comparisons. (C) Average ELISA absorbances across a dilution series of mouse sera for the TmENC control and E‐L2 experimental treatments. For both treatments, the ELISA plates were coated with synthetic L2 peptide to assess the epitope‐specific immune response. Pre‐immune bleeds (PI) are represented by dotted lines and final bleeds (FB) by solid lines. Error bars represent the standard deviation of the mean. E‐L2: FepA loop 2–TmENC variant; FepA: Ferric enterobactin receptor; TmENC: *Thermotoga maritima* encapsulin.

To further investigate the L2‐specific antibody responses from individual mice, we coated the ELISA plates with synthetic L2 peptide. In the final bleed, the TmENC control treatment induced an antibody response with an endpoint titer of 2000‐times dilution, while the E‐L2 experimental treatment induced an antibody response with an endpoint titer of 512 000‐times dilution (Fig. 1C). This provides further support that a significant antibody response induced in E‐L2 experimental mice is specific to FepA loop 2.

While we found a significant immune response to L2, we asked whether multiple sites within the TmENC subunit could be simultaneously modified for a broader and stronger immune response to multiple *Salmonella* epitopes. Based on the modification sites within the TmENC subunit (Fig. 2A,B) reported by Michel‐Souzy *et al*. [20], two L2 TmENC variants were designed, each with the insertion of L2 flanked by flexible linkers (G_4_S) between residues E61‐N62 and E124‐K125, respectively. Both variants contained a 6xHis‐tag flanked by flexible linkers at residues K239‐D240. A third variant was designed with L2 inserted between residues K239‐D240 and a 10xHis‐tag with a flexible linker added to the C terminus of the TmENC subunit. This was done to assess whether a C‐terminal His‐tag could be used for protein detection and purification, making position K239‐D240 available for peptide insertion, as we had previously inserted a 6xHis‐tag at this location for all TmENC variants assessed [19]. The TmENC variants are referred to as L2‐61, L2‐124, and L2‐239, respectively, and were assessed alongside the previously designed variant E‐L2 [19], referred to from here on as L2‐138 (Fig. 2C). L2‐61, L2‐124, and L2‐239 were codon optimized for nuclear expression in *N. benthamiana* and were cloned into plant expression vectors which enable protein targeting to the chloroplast and cytosol (Fig. 2D), as we previously achieved high accumulation levels of FepA TmEnc variants targeted to these subcellular locations [19].

**Fig. 2:**
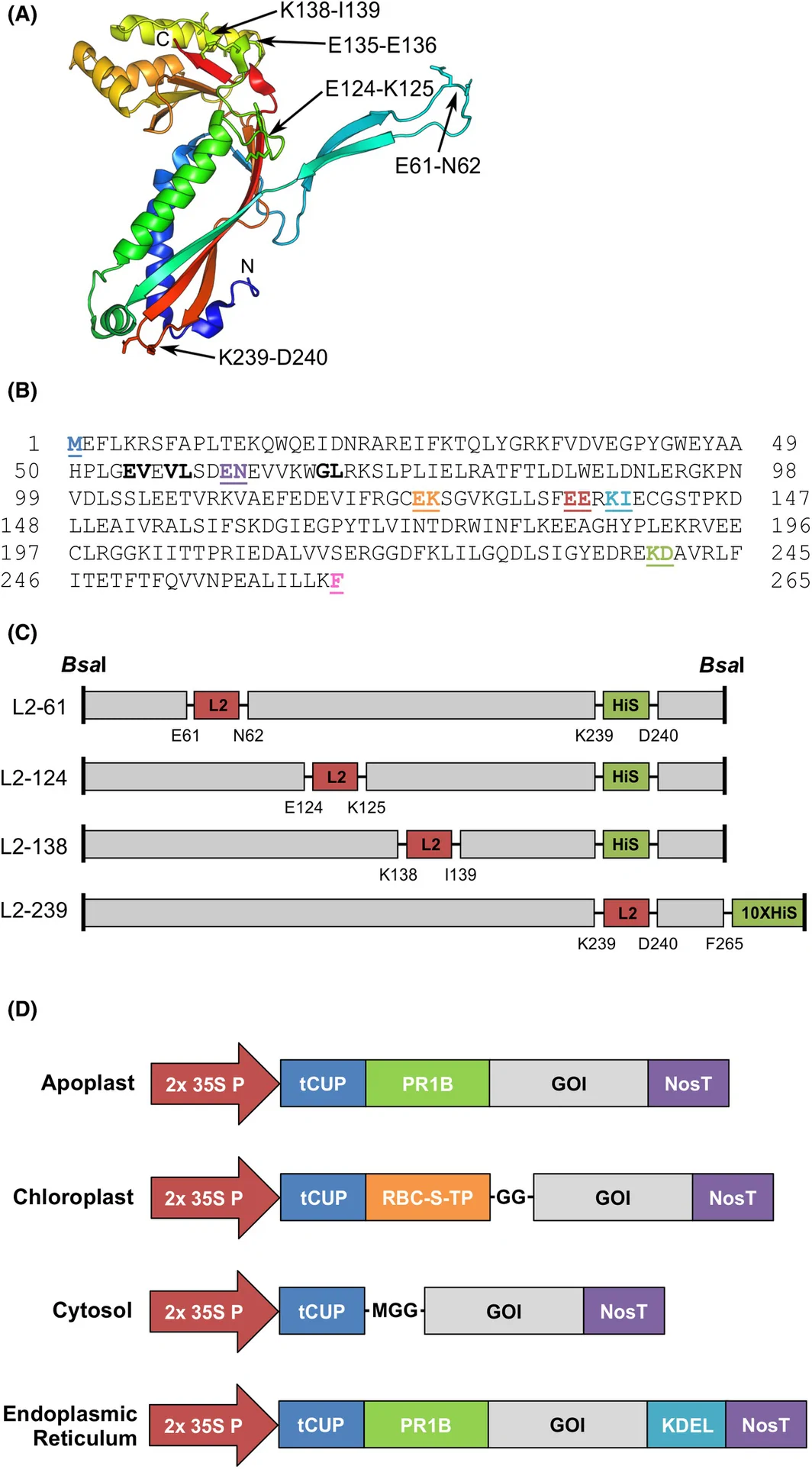
Modification sites within the *Thermotoga maritima* encapsulin subunit and design of plant expression vectors. (A) *Thermotoga maritima* encapsulin (TmENC) subunit with the C terminus (C), N terminus (N), and modifications sites utilized in this study (E61‐N62, E124‐K125, E135‐E136, K138‐I139, and K239‐D240) indicated. Structures generated using PyMOL (The PyMOL Molecular Graphics System). (B) Reference TmENC sequence retrieved from Protein Data Bank (PDB: 3DKT). The N‐terminal methionine (blue) was removed prior to gene synthesis as a start codon is encoded in the pCaMLite‐GoldenGate‐X (pCLGG‐X) plant expression vectors. The modification sites utilized in this study are indicated in purple (E61‐N42), orange (E124‐K125), red (E135‐E136), teal (K138‐I139), green (K239‐D240), and pink (C terminus; F265). All other modification sites are indicated in bold. (C) L2 TmENC variants containing the insertion of loop 2 (L2; red) from ferric enterobactin receptor (FepA) at sites E61‐N62 (L2‐61), E124‐K125 (L2‐124), K138‐I139 (L2‐138), and K239‐D240 (L2‐239). Gray bars represent the TmENC sequence. All the constructs contain a 6xHis‐tag (His; green) at site K239‐D240, except for L2‐239 which contains a 10xHis‐tag at the C terminus. L2 and the His‐tags are flanked by the linker sequence (G_4_S) to allow for flexibility (horizontal black lines). The constructs are flanked by *Bsa*I restriction enzyme sites (vertical black lines) for cloning into pCLGG‐X plant expression vectors. (D) pCLGG‐X plant expression vectors designed to target recombinant proteins to the chloroplast, cytosol, apoplast, or endoplasmic reticulum (ER). All vectors contain the double‐enhanced 35S promoter (red), tCUP translation enhancer (blue), gene insertion site (GOI; gray) and *Nos* terminator (purple). The transit peptide from the small subunit of RuBisCo (RBC‐S‐TP; orange) is included in the chloroplast vector to target the proteins to the chloroplast stroma. The apoplast and ER vectors contain a PR1b signal peptide (green) to target proteins to the secretory pathway. The ER vector contains a KDEL peptide (teal) for retrieval of the proteins to the ER. Cloning into the chloroplast vector results in the addition of two N‐terminal glycines (GG) to the mature TmENC proteins, while cloning into the cytosol vector results in the addition of a methionine and two glycines (MGG) to the N terminus.

For the cytosol‐targeted proteins, a single band was observed. The expected size for all the proteins is approximately 35 kDa; however, L2‐61 and L2‐239 ran slightly higher than L2‐124 and L2‐138 (Fig. 3A). All the constructs were sent for sequencing, and no errors were observed. It is possible that the size difference observed is due to a difference in conformation of these proteins when L2 is inserted closer to the termini (L2‐61 and L2‐239) versus the interior of the protein (L2‐124 and L2‐138). While the amino acid composition of these proteins is essentially the same, it is possible that the L2 sequence is interacting differently with the surrounding TmENC sequence based on its location within the subunit, potentially preventing complete denaturation of the proteins which could lead to the protein displaying higher electrophoretic mobility for L2‐124 and L2‐138 [42, 43]. Alternatively, the observed size difference may result from N‐terminal cleavage. L2‐138 was previously assessed alongside unmodified TmENC, where L2‐138 migrated only slightly above TmENC (expected size of 31 kDa) [19]. This suggests that modifications directed toward the middle of the TmENC subunit may promote partial N‐terminal cleavage.

**Fig. 3:**
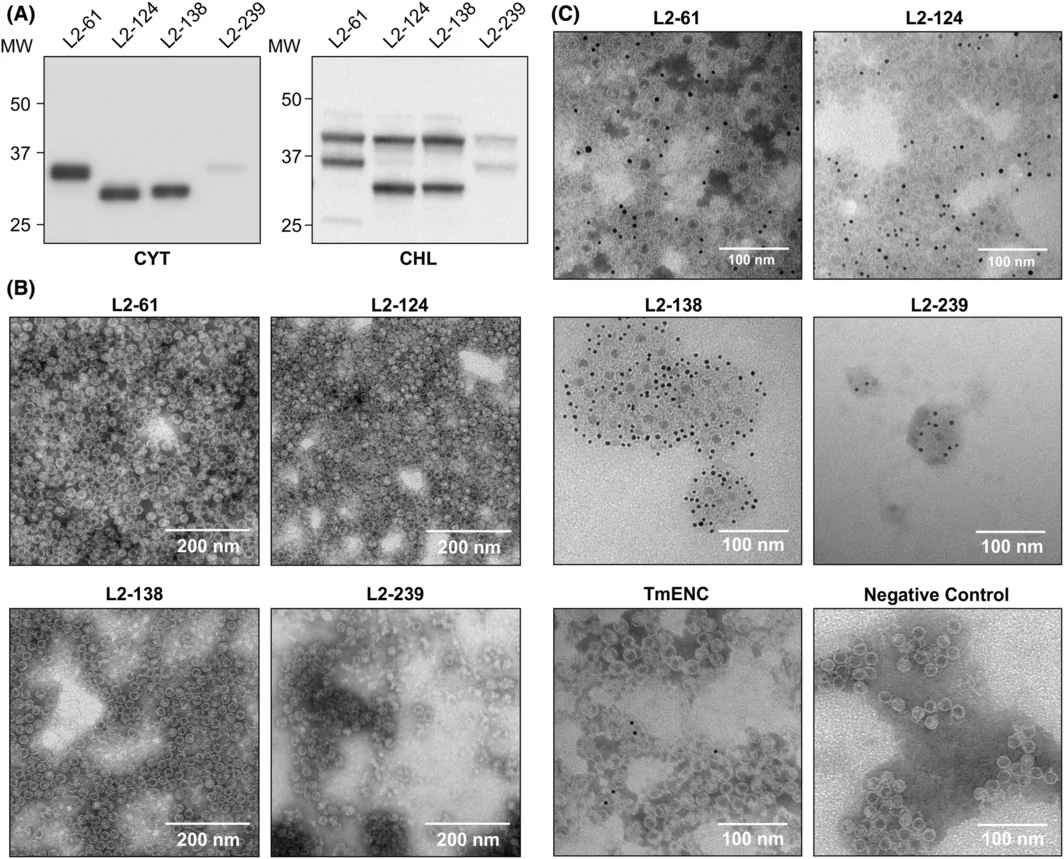
L2 TmENC variants accumulate and assemble in *N. benthamiana*. (A) The L2 TmENC variants (L2‐61, L2‐127, L2‐138. And L2‐239) were targeted to the chloroplast (CHL) and the cytosol (CYT) and leaf samples were collected on day 3 post‐infiltration. Lanes were loaded with total soluble protein extracted from 0.4 mg of L2‐61, L2‐127, and L2‐138 leaf tissue, and 4.0 mg of L2‐239 leaf tissue. Proteins were detected using an anti‐His (1 : 5000 dilution) primary antibody. The expected molecular weight of L2‐61, L2‐124, and L2‐138 is 35.5 kDa. The expected molecular weight of L2‐239 is 36.0 kDa. Results are representative of two replicates. MW: Molecular weight marker in kDa. (B) Negative‐stained transmission electron micrographs of purified L2 TmENC variants obtained using a JEOL JEM‐1400Flash (JEOL Ltd.) microscope operated at 80 kV. Images taken at 30000 × magnification. Scale‐bars represent 200 nm. (C) Purified unmodified TmENC and L2 TmENC variants were immunolabeled using an anti‐L2 primary antibody and a 6 nm gold‐conjugated secondary antibody, negatively stained, and examined using a JEOL JEM‐1400Flash (JEOL Ltd.) microscope operated at 80 kV. All images taken at 50000 × magnification. Scale‐bars represent 100 nm. (B, C) Images are representative of the entire grid and similar results were visualized across two replicates. L2: FepA loop 2; L2‐#: L2 TmENC variant with insertion of L2 at the respective amino acid position; TmENC: *Thermotoga maritima* encapsulin.

This interpretation is further supported by observations for the chloroplast‐targeted proteins (Fig. 3A). The band observed between 37 and 50 kDa likely represents incomplete cleavage of the N‐terminal chloroplast transit peptide, consistent with previous reports of chloroplast‐targeted TmENC variants [19]. Notably, this band migrates at a similar size across all L2 TmENC variants. In contrast, the size differences between variants are apparent in the lower bands, which likely correspond to the mature processed protein for L2‐61 and L2‐239 and potentially N‐terminally truncated proteins for L2‐124 and L2‐138 following transit peptide removal.

Regardless of subcellular targeting, all the proteins accumulated to relatively similar levels except for L2‐239, which accumulated to visibly lower levels. Previous studies have also reported instability of TmENC variants with modifications at the C terminus [14, 20]. For the other proteins, it appears that modification of each of the different sites does not have an impact on protein accumulation.

To assess whether modification of the different sites within the TmENC subunit impacts assembly and nanoparticle formation, the L2 TmENC variants were purified from infiltrated leaves using immobilized metal affinity chromatography (IMAC) followed by size‐exclusion chromatography (SEC). The purified nanoparticles were then visualized using transmission microscopy. Uniform round nanoparticles, approximately 22 nm in diameter, were observed for all the L2 TmENC variants (Fig. 3B). After proper assembly was confirmed, immunogold labeling was performed to assess whether the L2 epitope is displayed on the surface of assembled TmENC nanoparticles upon insertion at each of the modification sites. Using an anti‐L2 primary antibody, gold particles were observed near assembled nanoparticles for all the samples, confirming that the L2 epitope is surface‐exposed at each of the modification sites (Fig. 3C). We previously demonstrated that the anti‐L2 antibody does not bind unmodified TmENC via immunoblotting [19]; however, to further confirm its specificity, unmodified TmENC was immunolabeled as a control. Gold particles were observed but to a much lesser extent than with the L2 TmENC variants. No gold particles were observed in the negative control where the anti‐L2 primary antibody was omitted.

After determining that L2‐61, L2‐124, and L2‐239 express and assemble in *N. benthamiana*, we wanted to assess whether epitopes could be inserted simultaneously into multiple modification sites within the TmENC subunit without impeding nanoparticle assembly. If possible, this would have practical applications for vaccine development, potentially enabling the development of multivalent TmENC‐based vaccines.

We previously predicted the structure of FepA [19] and from this we selected candidate epitopes based on their antigenicity determined by the online prediction tools VaxiJen [44], SVMTriP [45], and Antigenic Peptide Prediction from the Immunomedicine Group (http://imed.med.ucm.es/Tools/antigenic.html). Additionally, a study by Newton *et al*. demonstrated that deletion of FepA loop 2, 10, or 11 reduced the affinity of FepA for iron, while deletions of loop 7 or 8 inhibited the ability of FepA to bind or transport iron [40]. Therefore, combining this with predicted antigenicity, FepA loops 2 (L2), 7 (L7), 10 (L10), and 11 (L11) were selected as the most suitable candidates (Table 1).

**Table 1:** Loops identified and utilized from the *Salmonella* iron acquisition proteins ferric enterobactin receptor (FepA), colicin receptor A (CirA), and salmochelin‐iron receptor (IroN).

Loops selected from *salmonella* iron acquisition proteins	Amino acid sequence
**FepA**	
Loop 2 (L2)	QADARNINQGHQSERTGSYADTLPAGRE
Loop 7 (L7)	APSLYQTNPNYILYSKGQGCYATGAGTGIGCYMMGNDDLKA
Loop 10 (L10)	GKQEPKKYDYQGNPVTGTDKQAVSP
Loop 11 (L11)	NLFDKRLWREGNAQTVRDTQTGAYMAGAGAYTYNEPGR
**CirA**	
Loop 7 (L7)	APSLLQLSPDWATNSCRGGCRIVGSPDLKP
Loop 8 (L8)	NRISISRTPDVNAAPGYSNFVGFETNSRGQRVPVFRY
Loop 10 (L10)	NVGDKDLKRDDYGYTEDGR
**IroN**	
Loop 5 (L5)	DPSSTSLTVKDSNIAGIPGSAANRSSK
Loop 8 (L8)	AGDQIIGRSASGAYVLQWQNGG
Loop 10 (L10)	GKQKPRTHAESRSEETKGLSGKALGA

Using modification sites E61‐N62, E124‐K125, E135‐E136, and K239‐D240, we designed two FepA TmENC variants: F3, which contains epitopes in all sites except for E124‐K125, and F4, which contains FepA epitopes in all four modification sites (Fig. 4A). For both constructs, the epitopes were flanked by flexible linkers and a 6xHis‐tag was connected to the epitope inserted in position K239‐D240 via a flexible linker. Both constructs were codon optimized for nuclear expression in *N. benthamiana* and F3 was cloned into chloroplast, cytosol, apoplast, and endoplasmic reticulum (ER) plant expression vectors (Fig. 2D) to assess which compartment would be most suitable for accumulation and assembly of the nanoparticles.

**Fig. 4:**
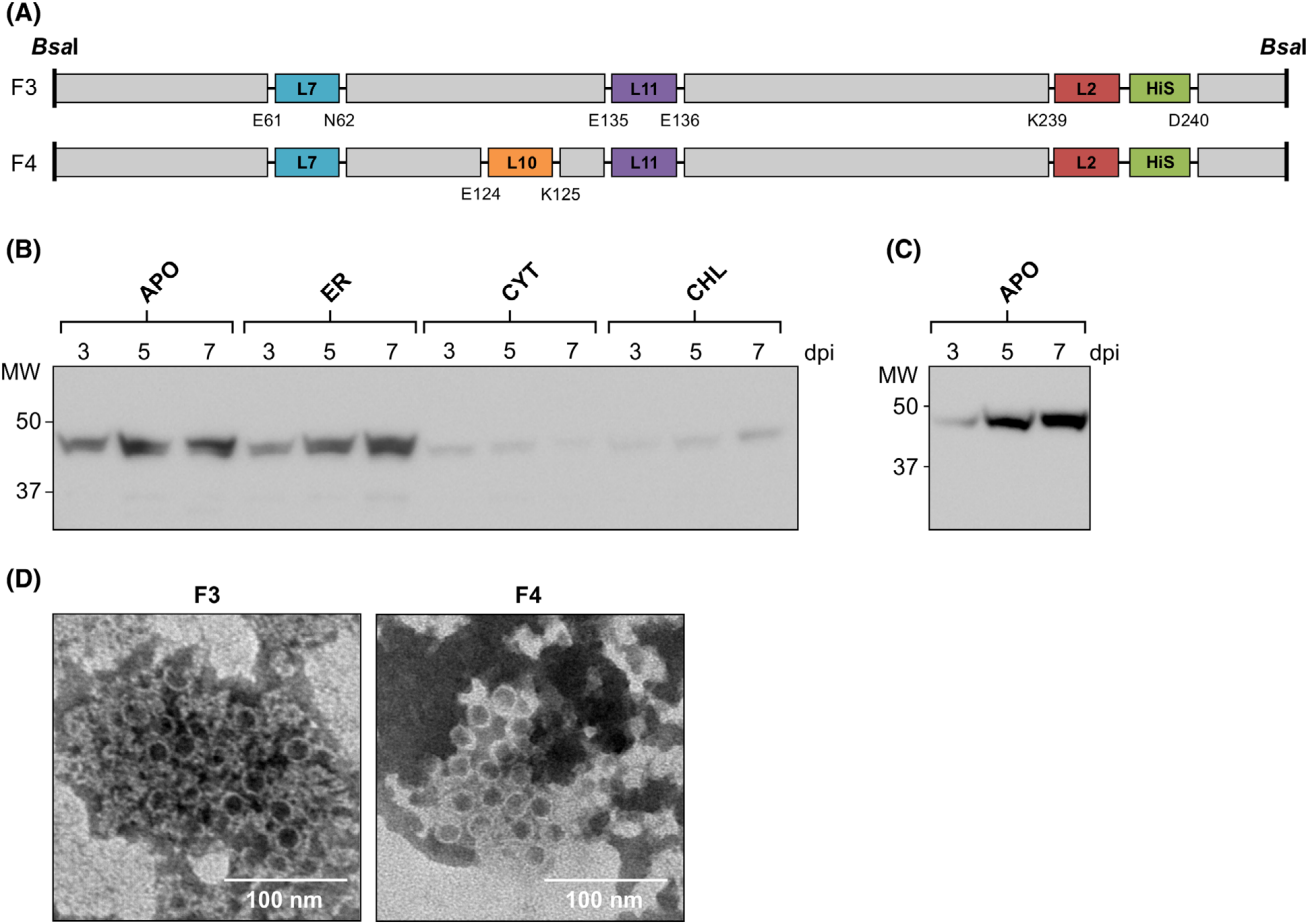
TmENC variants accumulate and assemble upon insertion of FepA epitopes at multiple sites. (A) FepA TmENC variant F3 containing the insertion of FepA loops 7 (L7; blue), 11 (L11; purple), and 2 (L2; red), at sites E61‐N62, E135‐E136, and K239‐D240, respectively, and FepA TmENC variant F4 containing the insertion of FepA loops 7 (L7; blue), 10 (L10; orange), 11 (L11; purple), and 2 (L2; red), at sites E61‐N62, E124‐K125, E135‐E136, and K239‐D240, respectively. Gray bars represent the TmENC sequence. A 6xHis‐tag (His; green) is also included at site K239‐D240 and all of the insertions are flanked by the linker sequence (G_4_S) to allow for flexibility (horizontal black lines). The constructs are flanked by *Bsa*I restriction enzyme sites (vertical black lines) for cloning into pCaMLite‐GoldenGate‐X (pCLGG‐X) plant expression vectors. (B) Accumulation of F3 over time and across different subcellular compartments. F3 was targeted to the apoplast (APO), endoplasmic reticulum (ER), cytosol (CYT), and chloroplast (CHL). Samples were collected on days 3, 5, and 7 post‐infiltration (dpi). The expected molecular weight of F3 is 45.0 kDa. (C) Accumulation of apoplast‐targeted (APO) F4 over time. Samples were collected on days 3, 5, and 7 post‐infiltration. The expected molecular weight of F4 is 48.3 kDa. (B, C) All lanes were loaded with total soluble protein extracted from 6.7 mg of leaf tissue. Proteins were detected using an anti‐His (1 : 5000 dilution) primary antibody. Results are representative of two replicates. MW: Molecular weight marker in kDa. (D) Negative‐stained transmission electron micrographs of purified F3 and F4 proteins obtained using a JEOL JEM‐1400Flash (JEOL Ltd.) microscope operated at 80 kV. Images taken at 40000 × magnification. Scale‐bars represent 100 nm. Images are representative of the entire grid and similar results were visualized across two replicates. FepA: Ferric enterobactin receptor; F3: FepA three epitope TmENC variant; F4: FepA four epitope TmENC variant; TmENC: *Thermotoga maritima* encapsulin.

All the F3 constructs were transiently expressed in *N. benthamiana* via agroinfiltration, and a time‐course experiment was conducted to determine on which day post‐infiltration accumulation reaches its peak. Unlike with previous fusion proteins, accumulation of F3 was very low in both the chloroplast and cytosol, while higher accumulation was observed in the apoplast and ER (Fig. 4B). Based on these results, we chose to clone F4 into the apoplast and ER vectors only. Despite multiple attempts, cloning of F4 into the ER vector was unsuccessful; however, the protein accumulated relatively well in the apoplast (Fig. 4C). Therefore, we decided to move forward with apoplast‐targeted expression for both proteins. Accumulation in the apoplast appeared to increase over time; therefore, samples were collected on day 7 post‐infiltration for all future experiments.

The quaternary assembly and nanoparticle formation of F3 and F4 were then assessed to determine whether the insertion of multiple epitopes within the TmENC subunit impedes nanoparticle assembly. For this, F3 and F4 were purified from infiltrated plant tissue using IMAC and SEC. The purified samples were visualized using transmission electron microscopy and assembled nanoparticles were observed for both proteins (Fig. 4D), indicating that the simultaneous insertion of epitopes at different modification sites within the TmENC subunit does not impede nanoparticle assembly.

To assess whether the insertion of multiple epitopes within the TmENC subunit has an impact on recombinant protein accumulation, the accumulation of chloroplast‐targeted L2‐138 and apoplast‐targeted F3 and F4 was assessed by semi‐quantitative immunoblotting (Fig. 5A). The average accumulation level of L2‐138 (0.338 mg/g FW) was significantly higher (*P*‐value < 0.01) than that of F3 (0.012 mg/g FW) and F4 (0.015 mg/g FW) (Fig. 5B). This suggests that as additional peptides are added to the TmENC subunit, recombinant protein accumulation decreases. Thus, simultaneous modification of multiple sites within the TmENC subunit likely has a negative impact on protein stability.

**Fig. 5:**
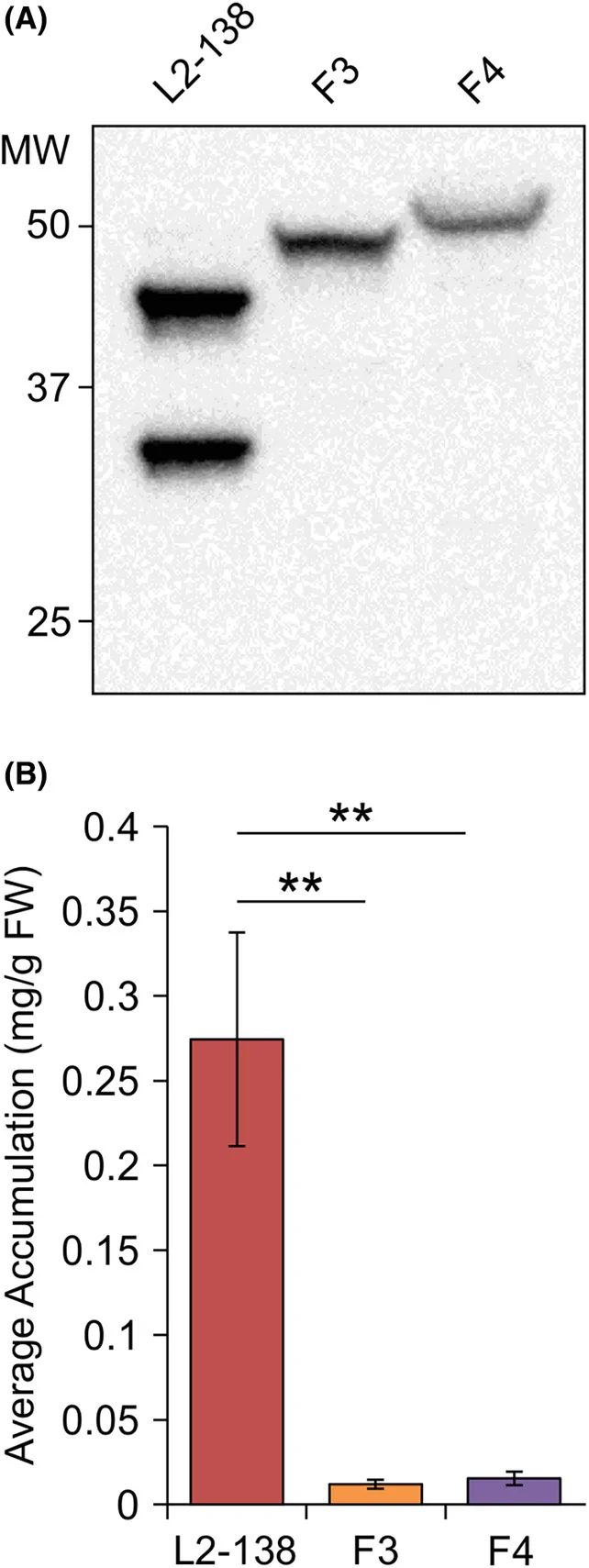
Increasing number of epitope insertions has a negative impact on the accumulation of FepA TmENC variants. (A) L2‐138, F3, and F4 were transiently expressed in *N. benthamiana* and leaf samples were collected on day 7 post‐infiltration. L2‐138 was targeted to the chloroplast and F3 and F4 were targeted to the apoplast. Lanes were loaded with total soluble protein extracted from 0.8 mg of L2‐138, and 8.0 mg of F3 and F4 leaf tissue, respectively. Proteins were detected using an anti‐His primary antibody (1 : 5000 dilution). Results are representative of two replicates. (B) Average accumulation levels for chloroplast‐targeted L2‐138 (*n* = 4) and apoplast‐targeted F3 (*n* = 5) and F4 (*n* = 5). All samples were collected on day 7 post‐infiltration. Significance (** = *P‐*value < 0.01) was determined using a one‐tailed Student's *t*‐test for unequal variance followed by a Benjamini‐Hochberg correction for multiple comparisons. Error bars represent the standard error of the mean. MW: Molecular weight marker in kDa. FepA: Ferric enterobactin receptor; F3: FepA three epitope TmENC variant; F4: FepA four epitope TmENC variant; FW: fresh leaf weight; L2: FepA loop 2; L2‐138: L2 TmENC variant with L2 insertion at amino acid position 138; TmENC: *Thermotoga maritima* encapsulin.

Alongside F3 and F4, we also designed and assessed two TmENC variants containing epitopes from CirA and IroN. The structures of CirA and IroN have not been solved; therefore, structure prediction was performed for these proteins as previously described for FepA [19], and candidate epitopes were selected based on their antigenicity determined through the online prediction tools mentioned above. For CirA, three candidate epitopes, loops 7 (L7), 8 (L8), and 10 (L10), were identified, and for IroN, the three best candidates were loops 5 (L5), 8 (L8), and 10 (L10) (Table 1). Using these epitopes, CirA (C3) and IroN (I3) TmENC variants were designed with the epitopes inserted at sites E61‐N62, E135‐E136, and K239‐D240 (Fig. 6A). For both variants, the epitopes were flanked by flexible linkers, and a 6xHis‐tag was connected to the epitope inserted in site K239‐D240 via a flexible linker. Both variants were codon optimized for nuclear expression in *N. benthamiana*. C3 was cloned into all four plant expression vectors, while I3 was only cloned into the chloroplast vector. I3 contains one potential glycosylation site; therefore, we chose to avoid the secretory pathway and targeted I3 to the chloroplast.

**Fig. 6:**
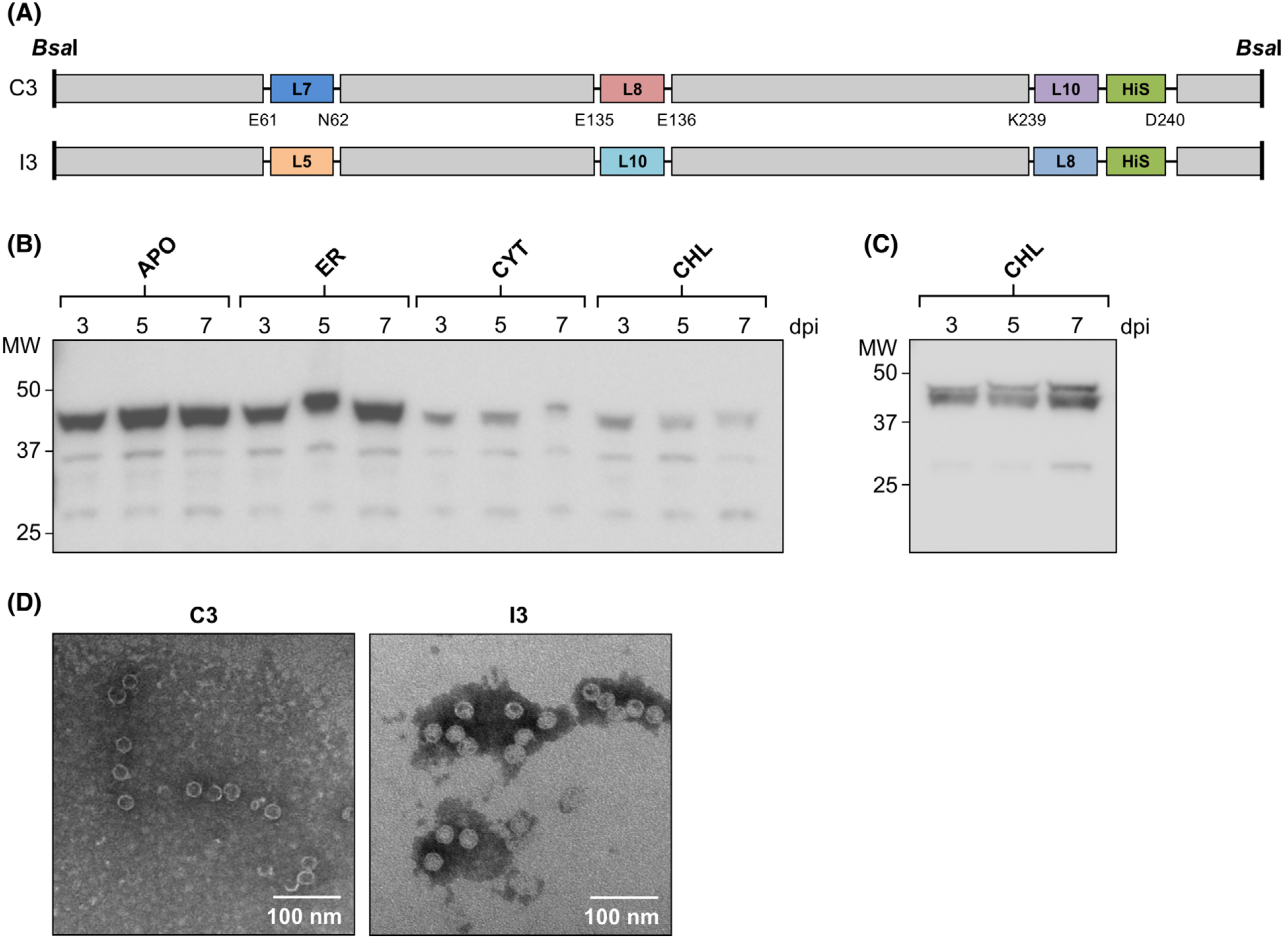
CirA and IroN TmENC variants accumulate and assemble in *N. benthamiana*. (A) CirA TmENC variant C3 containing the insertion of CirA loops 7 (L7; dark blue), 8 (L8; pink), and 10 (L10; purple), at sites E61‐N62, E135‐E136, and K239‐D240, respectively. IroN TmENC variant I3 containing the insertion of IroN loops 5 (L5; orange), 10 (L10; teal), and 8 (L8; light blue), at sites E61‐N62, E135‐E136, and K239‐D240, respectively. Gray bars represent the TmENC sequence. A 6xHis‐tag (His; green) is also included at site K239‐D240 and all the insertions are flanked by the linker sequence (G_4_S) to allow for flexibility (horizontal black lines). The constructs are flanked by *Bsa*I restriction enzyme sites (vertical black lines) for cloning into pCaMLite‐GoldenGate‐X (pCLGG‐X) plant expression vectors. (B) Accumulation of C3 over time and across different subcellular compartments. C3 was targeted to the apoplast (APO), endoplasmic reticulum (ER), cytosol (CYT), and chloroplast (CHL). Samples were collected on days 3, 5, and 7 post‐infiltration (dpi). All lanes were loaded with total soluble protein extracted from 6.7 mg of leaf tissue. The expected molecular weight of C3 is 42.9 kDa. (C) Accumulation of chloroplast‐targeted (CHL) I3. Samples were collected on days 3, 5, and 7 post‐infiltration and all lanes were loaded with total soluble protein extracted from 3.3 mg of leaf tissue. The expected molecular weight of I3 is 41.0 kDa. (B, C) Proteins were detected using an anti‐His (1 : 5000 dilution) primary antibody. Results are representative of two replicates. MW: Molecular weight marker in kDa. (D) Negative‐stained transmission electron micrographs of purified C3 and I3 proteins obtained using a JEOL JEM‐1400Flash (JEOL Ltd.) microscope operated at 80 kV. Images taken at 40000 × magnification. Scale‐bars represent 100 nm. Images are representative of the entire grid and similar results were visualized across two replicates. CirA: colicin receptor A; C3: CirA three epitope TmENC variant; IroN: Salmochelin‐iron receptor; I3: IroN three epitope TmENC variant; TmENC: *Thermotoga maritima* encapsulin.

To evaluate the expression of C3, all of the constructs were transiently expressed in *N. benthamiana* and a time‐course was conducted. A band of approximately 43 kDa corresponds to the expected size for C3 while the two faint lower bands (< 37 kDa) are likely the result of degradation (Fig. 6B). Relatively high accumulation levels were observed for the apoplast‐targeted protein, with levels appearing to increase gradually over time. Thus, for all further experiments, C3 was targeted to the apoplast and samples were collected on day 7 post‐infiltration.

For chloroplast‐targeted I3, the protein accumulated well with levels increasing slightly as time progressed (Fig. 6C), therefore, for all further experiments, samples were collected on day 7 post‐infiltration. A double‐banding pattern was observed, with the lower band, approximately 41 kDa, corresponding to the expected size of the mature monomeric protein. As described previously with the E‐L2 TmENC variant [19], the upper band likely represents the immature protein prior to removal of the chloroplast transit peptide.

To confirm that C3 and I3 assemble into nanoparticles, both proteins were purified via IMAC and SEC and visualized using transmission electron microscopy. As with F3 and F4, nanoparticles were observed for both C3 and I3 (Fig. 6D), providing further support that modification of multiple sites within the TmENC subunit does not impede nanoparticle assembly.

To compare accumulation levels of the mono‐, tri‐, and quadrivalent variants we used the highest expressing constructs for each variant: chloroplast‐targeted TmENC, L2‐138, and I3, and apoplast‐targeted C3, F3, and F4 (Fig. 7). All of the tri‐ and quadrivalent variants accumulated to statistically significantly lower levels (*P*‐value < 0.05) than the monovalent L2‐138 variant, while all but I3 accumulated to significantly lower levels (*P*‐value < 0.05) than TmENC containing only a 6XHis‐tag at position K239‐D240. The accumulation of I3 (0.164 mg/g FW) was significantly higher (*P*‐value < 0.05) than F3 (0.012 mg/g FW), F4 (0.015 mg/g FW), and C3 (0.024 mg/g FW), which accumulated to much lower levels. The low yields observed for F3, F4, and C3 suggest that simultaneously modifying multiple sites within the TmENC subunit negatively impacts protein stability, resulting in decreased accumulation. However, the higher yield of I3 suggests that protein stability and accumulation may be influenced not only by the number of modifications but also by their specific nature.

**Fig. 7:**
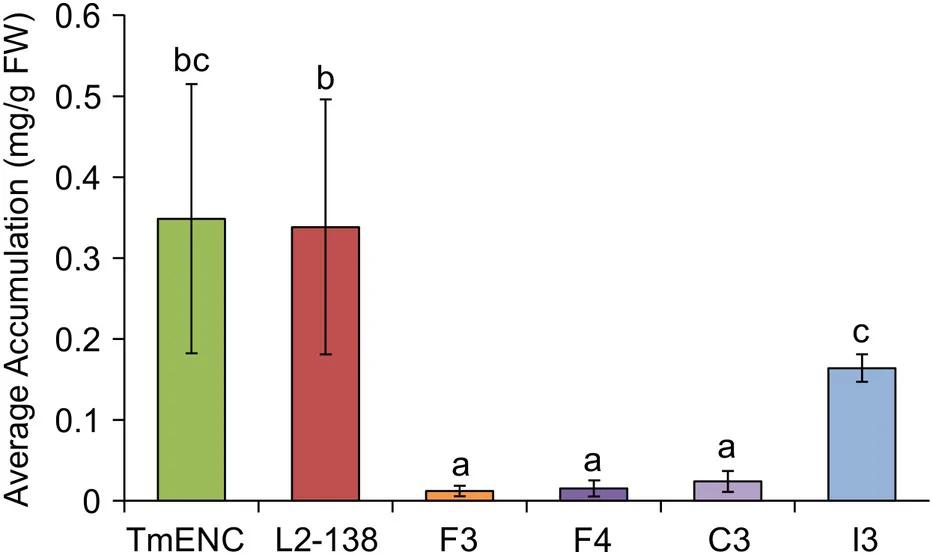
Accumulation of TmENC variants. Average accumulation of TmENC and mono‐ (L2‐138), tri‐ (C3, F3, I3), and quadrivalent (F4) TmENC variants. TmENC, L2‐138, and I3 were targeted to the chloroplast, and C3, F3, and F4 were targeted to the apoplast. All samples were collected on day 7 post‐infiltration. Significance was determined using a one‐tailed Student's *t*‐test for unequal variance, comparing means for all groups, followed by a Benjamini‐Hochberg correction for multiple comparisons. Error bars represent the standard error of the mean. Letters denote significance groupings (*P‐*value < 0.05). Results are representative of two replicates (*n* = 5 for all proteins). CirA: colicin receptor A; C3: CirA three epitope TmENC variant; FepA: Ferric enterobactin receptor; F3: FepA three epitope TmENC variant; F4: FepA four epitope TmENC variant; FW: fresh leaf weight; IroN: Salmochelin‐iron receptor; I3: IroN three epitope TmENC variant; L2‐138: L2 TmENC variant with genetic fusion at amino acid position 138; TmENC: *Thermotoga maritima* encapsulin.

Building on this observation, we hypothesized that the nature of the inserted sequences plays a more critical role than the overall number of insertions. To further investigate this, we designed two additional CirA TmENC variants: C1 with one epitope, loop 8 (L8), inserted at modification site E135‐E136, and C2 with two epitopes, loop 7 (L7) and loop 10 (L10), at sites E135‐E136 and K239‐D240, respectively (Fig. 8A). For C2, a 6xHis‐tag was connected to the epitope inserted in site K239‐D240 via a flexible linker, while for C1, site K239‐D240 contained only a 6xHis‐tag flanked by flexible linkers. Both constructs were cloned into the apoplast‐targeted plant expression vector, as apoplast‐targeted C3 had the highest accumulation out of all of the subcellular locations assessed (Fig. 6B).

**Fig. 8:**
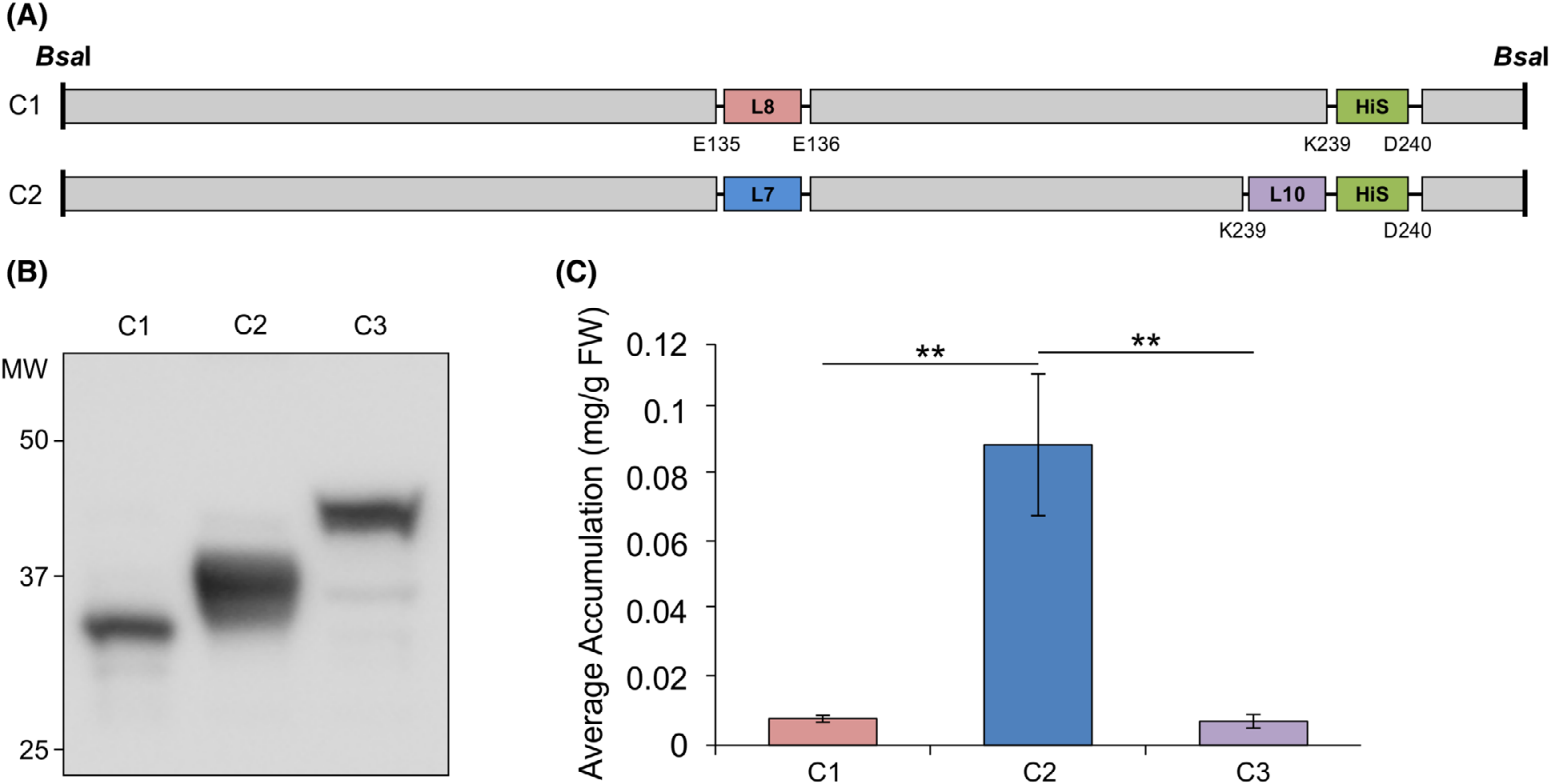
Accumulation of CirA TmENC variants varies with number of epitope insertions. (A) CirA TmENC variants C1, containing the insertion of CirA loop 8 (L8; pink) at site E135‐E136, and C2, containing the insertion of CirA loops 7 (L7; blue) and 10 (L10; purple) at sites E135‐E136 and K239‐D240, respectively. Gray bars represent the TmENC sequence. A 6xHis‐tag (His; green) is also included at site K239‐D240 and all of the insertions are flanked by the linker sequence (G_4_S) to allow for flexibility (horizontal black lines). The constructs are flanked by *Bsa*I restriction enzyme sites (vertical black lines) for cloning into pCaMLite‐GoldenGate‐X (pCLGG‐X) plant expression vectors. (B) Apoplast‐targeted C1, C2, and C3 were transiently expressed in *N. benthamiana*, and leaf samples were collected on day 7 post‐infiltration. Lanes were loaded with total soluble protein extracted from 4.0 mg of leaf tissue and proteins were detected using an anti‐His primary antibody (1 : 5000 dilution). The expected molecular weights of C1, C2, and C3 are 36.5, 38.1, and 42.9 kDa, respectively. Results are representative of two replicates. MW: Molecular weight marker in kDa. (C) Average accumulation levels for apoplast‐targeted C1, C2, and C3 (*n* = 9 for all proteins). Significance (** = *P‐*value < 0.01) was determined using a one‐tailed Student's *t*‐test for unequal variance followed by a Benjamini‐Hochberg correction for multiple comparisons. Error bars represent the standard error of the mean. CirA: colicin receptor A; C1: CirA single epitope TmENC variant; C2: CirA two epitope TmENC variant; C3: CirA three epitope TmENC variant; FW: fresh leaf weight; TmENC: *Thermotoga maritima* encapsulin.

To compare the accumulation levels of the CirA TmENC variants, apoplast‐targeted C1, C2, and C3 were transiently expressed in *N. benthamiana* and their accumulation levels were assessed using semi‐quantitative immunoblotting (Fig. 8B). The average accumulation level of C2 (0.090 mg/g FW) was significantly higher (*P*‐value < 0.01) than that of both C1 (0.008 mg/g FW) and C3 (0.007 mg/g FW) (Fig. 8C). C1 and C3 both contain CirA loop 8 (L8) within site E135‐E136, while L8 was excluded from C2. These results suggest that the presence of L8 within site E135‐E136 negatively impacts protein stability and accumulation, and thus provide further support that the nature of the peptide insertions, not the number, within the TmENC subunit influence protein stability and accumulation.

To investigate why the presence of CirA L8 has a negative effect on protein stability and yield, we utilized DNASTAR Protean 3D to analyze the peptide sequence, net charge, and average hydropathy for all the FepA, CirA, and IroN loops (Table 2). Out of the three CirA loops utilized (L7, L8, and L10), L8 is the largest at 37 amino acids, while L7 and L10 are 30 and 19 amino acids, respectively. FepA L7 and FepA L11 are comparable in size to CirA L8, 41 and 38 amino acids, respectively, and F3 and F4, which contain both of these loops, accumulated to similar levels as C1 and C3 which contain CirA L8 (Fig. 7). This suggests that large insertions may negatively impact the stability of the TmENC subunit.

**Table 2:** Physiochemical characteristics of the loops utilized from the *Salmonella* iron acquisition proteins ferric enterobactin receptor (FepA), colicin receptor A (CirA), and salmochelin‐iron receptor (IroN).

Loops selected from *salmonella* iron acquisition proteins	Peptide length (amino acids)	Net charge (at pH = 7.0)	Average Hydropathy
**FepA**			
Loop 2 (L2)	28	−0.91	−1.42
Loop 7 (L7)	41	−0.13	−0.3
Loop 10 (L10)	25	1	−1.63
Loop 11 (L11)	38	0.99	−1.03
**CirA**			
Loop 7 (L7)	30	0.87	−0.23
Loop 8 (L8)	37	2.98	−0.61
Loop 10 (L10)	19	−2.01	−1.99
**IroN**			
Loop 5 (L5)	27	1	−0.55
Loop 8 (L8)	22	0	−0.25
Loop 10 (L10)	26	3.09	−1.38

In addition to its large size, CirA L8 also has a high positive net charge of 2.98 and a moderate average hydropathy score of −0.61. In comparison, IroN L10 is smaller (26 amino acids), has a high positive net charge (3.09), and a low average hydropathy score (−1.38). I3 accumulated to significantly higher levels than C3, indicating that loop size and perhaps the hydropathy score are more important determinants of protein stability than the net charge of the loops.

We then asked whether a loop with fewer than 30 amino acids, a low net charge, and a low average hydropathy score could be inserted in the three identified positions in TmENC without impacting accumulation of the modified nanoparticle. FepA L2 is 28 amino acids in length, has a net charge of −0.91, and an average hydropathy score of −1.42, and individual insertions of L2 at positions E61‐N62, E124‐K125, and K138‐I139 did not have an impact on protein accumulation (Fig. 3A). Therefore, we designed an additional L2 TmENC variant, L2X3, with L2 inserted in sites E61‐N62, E135‐E136, and K239‐D240, as in the other trivalent variants (Fig. 9A). L2X3 was cloned into the cytosol‐targeted plant expression vector.

**Fig. 9:**
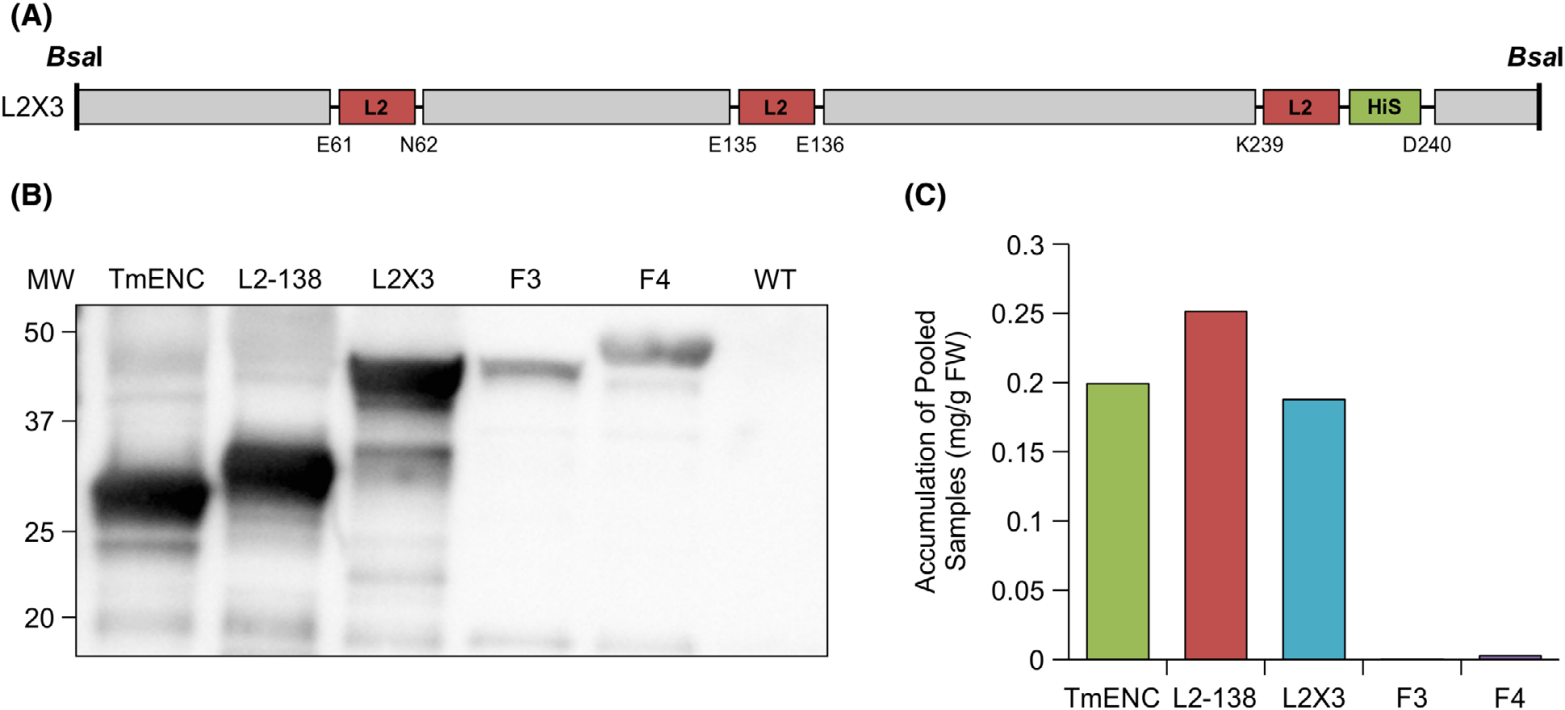
Trivalent TmENC variant L2X3 accumulates to similar levels as the monovalent variant L2‐138. (A) L2 TmENC variant L2X3 containing the insertion of FepA loop 2 (L2; red) at sites E61‐N62, E135‐E136, and K239‐D240, respectively. Gray bars represent the TmENC sequence. A 6xHis‐tag (His; green) is also included at site K239‐D240 and all the insertions are flanked by the linker sequence (G4S) to allow for flexibility (horizontal black lines). The construct is flanked by *Bsa*I restriction enzyme sites (vertical black lines) for cloning into pCaMLite‐GoldenGate‐X (pCLGG‐X) plant expression vectors. (B) Cytosol‐targeted TmENC, L2‐138, L2X3, F3, and F4 were transiently expressed in *N. benthamiana*, and leaf samples were collected on day 5 post‐infiltration. Lanes were loaded with total soluble protein extracted from 2.0 mg of leaf tissue and proteins were detected using an anti‐His primary antibody (1 : 5000 dilution). The expected molecular weights of TmENC, L2‐138, L2X3, F3, and F4 are 31.7, 35.5, 42.7, 45.0, and 48.3 kDa, respectively. Results are representative of two replicates. MW: Molecular weight marker in kDa. WT: Soluble protein extract from wild‐type *N. benthamiana* tissue. (C) Accumulation levels of pooled samples from five biological replicates for cytosol‐targeted proteins. FepA: Ferric enterobactin receptor; F3: FepA three epitope TmENC variant; F4: FepA four epitope TmENC variant; FW: fresh leaf weight; L2: FepA loop 2; L2‐138: L2 TmENC variant with L2 insertion at amino acid position 138; L2X3: L2 TmENC variant with L2 inserted at three distinct amino acid positions; TmENC: *Thermotoga maritima* encapsulin.

Cytosol‐targeted L2X3 (0.1878 mg/g FW) accumulated to similar levels as cytosol‐targeted TmENC (0.1994 mg/g FW) and L2‐138 (0.2513 mg/g FW), while lower accumulation was observed for cytosol‐targeted F3 (0.0002 mg/g FW) and F4 (0.0029 mg/g FW), which both contain L2 at position K239‐D240, and also FepA loops 7 and 11 which are larger in size (Fig. 9B,C). This provides further evidence that the physiochemical properties of the insertions, not the number of insertions within the TmENC subunit, influence TmENC stability and accumulation.

## Discussion

In the pursuit to develop effective vaccines, antigen presentation ensuring high immunogenicity is crucial [46, 47]. PNPs have become attractive candidates for antigen display due to their biocompatibility, high stability, and manipulability, and enhanced immunogenicity through the efficient delivery to antigen‐presenting cells [1, 6, 48, 49]. The repetitive subunit organization of PNPs enables high‐density display of antigens on their surface, effectively promoting immune recognition, and their large supramolecular structure can confer adjuvant‐like properties resulting in a robust immune response [1]. Additionally, PNPs can promote antigen presentation within mucosa‐associated lymphoid tissue, leading to enhanced mucosal immune responses. They have also been shown to maintain both mucosal immune balance and epithelial homeostasis by regulating inflammation and transiently opening epithelial tight junctions [50]. Thus, PNPs are attractive platforms for overcoming the fundamental challenge of developing effective vaccines and represent a promising strategy for the development of an effective oral *Salmonella* vaccine for poultry.

While various types of natural and synthetic PNPs have been utilized as platforms for vaccine development [1, 3, 4, 5, 6], here we have utilized encapsulin from *T. maritima* (TmENC) [15]. Numerous studies have investigated the potential of TmENC as an antigen display platform, utilizing different modification sites within the subunit [12, 29, 51]. Lagoutte *et al*. genetically fused the ectodomain of Matrix protein 2 from influenza A within an internal surface‐exposed site [12], while Jung *et al*. fused the core region of the rotavirus VP8 protein to the C terminus (ENC‐P2‐VP8*) [29]. In both cases, immunization of mice with the engineered TmENC nanoparticles elicited antigen‐specific antibody responses. Markedly, ENC‐P2‐VP8* induced high virus neutralization titers, higher than those induced by P2‐VP8* without ENC [29]. Thus, these results confirm the potential of engineered TmENC nanoparticles for antigen display to address various health challenges.

Many studies have highlighted the antigenic potential of FepA and its extracellular loops [52, 53, 54, 55, 56, 57]. However, to our knowledge, we are the first to evaluate the immunogenicity of an individual FepA loop *in vivo*. Previous studies have demonstrated that TmENC nanoparticles displaying antigens improve immunogenicity compared to the antigen alone. The 20–24 nm size range of TmENC nanoparticles facilitates efficient transport to the lymph nodes where they are captured and presented by dendritic cells and macrophages, amplifying immune activation [49, 58]. Thus, our approach of displaying *Salmonella* epitopes on TmENC is likely more effective than the administration of FepA epitopes on their own.

While a significant immune response was induced against FepA loop 2, the *Salmonella* iron acquisition proteins FepA, IroN, and CirA are functionally redundant and it has been suggested that a more significant protective effect may be observed if chickens are immunized with a vaccine containing all three proteins [27, 57]. For this reason, we produced FepA‐, IroN‐, and CirA TmENC variants that can be mixed together in the vaccine candidate.

Numerous studies have investigated different modification sites within the TmENC subunit; however, to our knowledge, no group has attempted modification of more than two sites for multivalent display of vaccine epitopes. One key advantage of displaying epitopes on large, multi‐subunit PNPs, like encapsulins, is defined and repetitive display [7, 20]. Modification of a single site within the TmENC subunit enables well‐defined surface display of 60 functional groups. This can be increased to up to 240 functional groups when each subunit has four different modifications.

With the monovalent L2 TmENC variants, we found that upon insertion of FepA L2 at four distinct sites within the TmENC subunit (E61‐N62, E‐124‐K125, K138‐I139, and K239‐D240), the L2‐124 and L2‐138 variants displayed higher electrophoretic mobility, suggesting either incomplete denaturation due to conformation differences of some variants [42, 43], or partial N‐terminal cleavage. Despite this, all the L2 TmENC variants correctly assembled into nanoparticles. TmENC nanoparticle assembly is primarily driven by conserved inter‐subunit interactions within the HK97‐like capsid fold, rather than by the extreme N‐terminal residues [15, 59]. Because the N terminus is oriented toward the nanoparticle interior and mainly contributes to internal architecture and cargo‐peptide interactions, moderate N‐terminal truncations can be tolerated while still permitting correct nanoparticle assembly [60].

Multivalent or multicomponent vaccines have the potential to provide broader and enhanced protection compared to their monovalent counterparts [61]. Key benefits of multivalent vaccines are broader immune coverage, reduced immune escape, and enhanced immunogenicity [62, 63, 64, 65]. The increased breadth of antigen exposure can lead to broader immune coverage by exposing the immune system to diverse strains or variants of a pathogen, or in our case, multiple antigens (i.e. multiple iron acquisition proteins), or multiple parts of one antigen (i.e. epitopes from a single iron acquisition protein), from the same pathogen. This expands the pool of immune targets and increases the likelihood of effective recognition. When responses are directed against different regions of a single protein, the resulting antibodies can act synergistically, enhancing neutralization or disrupting the protein's function more effectively than responses to a single epitope alone [66, 67, 68, 69]. While monovalent vaccines can fail if a mutation occurs in the target antigen, exposure to multiple antigens makes it harder for the pathogen to escape immunity, as it would require simultaneous mutations in several regions and several proteins [70]. Thus, a multivalent vaccine has the potential to induce a broader response, leading to longer‐lasting and more resilient protection.

With the design of the multivalent TmENC variants, our aim is to induce immune recognition and targeting of multiple regions of the iron acquisition proteins FepA, CirA, and IroN to produce an effective *Salmonella* vaccine for poultry. Formulating a vaccine that combines multivalent FepA, CirA, and IroN TmENC nanoparticles would provide two layers of multivalency by presenting multiple epitopes across multiple antigens. This approach could elicit a broader and more protective immune response and inhibit bacterial iron uptake.

Comparison of FepA mono‐ (L2‐138), tri‐ (F3), and quadrivalent (F4) TmENC variants revealed that while average accumulation of both F3 and F4 was significantly lower than that of L2‐138, their accumulation was not significantly different from each other. This indicates that, in the case of these FepA TmENC variants, increasing the number of modifications to produce quadrivalent variants does not have a significant impact on average recombinant protein accumulation. However, this was not the case with the CirA mono‐ (C1), bi‐ (C2), and trivalent (C3) variants, where there was no significant difference between the average accumulation of C1 and C3, while accumulation of C2 was significantly higher. In this case, it appears that the nature of the modifications, not the number, is impacting recombinant protein accumulation, as both C1 and C3 contain CirA loop 8 (L8) while C2 does not.

Out of the three CirA epitopes utilized, L8 is the largest. Larger peptides are bulkier and therefore may be more likely to interfere with the folding/assembly of TmENC. In similar particle systems, insertion of foreign peptides has been shown to destabilize folding and reduce assembly efficiency, with larger insertions being particularly disruptive to protein stability and multimeric particle assembly [71, 72, 73]. L8 also has a high positive net charge. Highly positive peptides have the potential to interfere with the folding of TmENC by forming electrostatic interactions with negatively charged residues within the protein, disrupting the folding energy landscape and stability of the native structure [74]. In addition to this, L8 is moderately hydrophobic. A high net positive charge combined with hydrophobic patches is known to correlate with protein insolubility and aggregation, and produces amphipathic regions that can interact strongly with negatively charged surfaces such as membranes and acidic regions on partially folded proteins. This can disrupt protein folding, promote aggregation, and accelerate degradation [75, 76, 77]. All of these properties could contribute to the low accumulation of TmENC variants containing L8.

Our hypothesis that the physiochemical properties of the insertions, not the number, influence recombinant protein accumulation is further supported by our results with the L2 TmENC variant L2X3. L2X3, which contains FepA L2 inserted in three distinct sites within the TmENC subunit, accumulated to similar levels as L2‐138 which contains one L2 insertion and TmENC with no insertions. Our results with CirA L8 and FepA L2 highlight that careful consideration should be taken when selecting epitopes and the sites within the TmENC subunit in which they will be inserted. Bioinformatic tools should be implemented in the design of multivalent nanoparticle vaccines to analyze the biochemistry and compatibility of the selected antigens/epitopes and the modification sites in which they will be inserted to avoid steric hinderance and structural strain that may negatively impact protein stability and accumulation [5, 78, 79].

While further work can be done to optimize the design of our multivalent TmENC variants for improved stability and accumulation, this study demonstrates that plant‐produced TmENC nanoparticles are immunogenic and provides a proof‐of‐concept for the use of TmENC for tri‐ and quadrivalent vaccine production through simultaneous modification of multiple sites within the TmENC subunit. Further studies will be required to assess the efficacy of our multivalent TmENC variants at preventing *Salmonella* colonization in poultry. Our long‐term goal is oral administration of lyophilized leaves containing these nanoparticles to induce a mucosal immune response at the site of *Salmonella* colonization in the gut. While we have produced TmENC variants containing epitopes from *Salmonella*, this strategy could be adapted to a wide range of pathogens. If successful, this could pave the way for the development of multivalent TmENC‐based vaccines for a variety of diseases.

## Materials and methods

### Ethics statement

All mouse immunization procedures were conducted at Cedarlane Labs (Burlington, ON, Canada) under the approved Animal Use Protocol AUP117AB. This protocol was reviewed and approved by Cedarlane's institutional Animal Care Committee and was developed in accordance with the Canadian Council on Animal Care (CCAC) guidelines for the ethical use of animals in research.

All mice used in the study were bred at the Cedarlane facility. Animal housing and handling followed procedures outlined in the CCAC guidelines. Briefly, a maximum of four mice were housed per cage and were provided food and water *ad libitum*. The mice were housed in a temperature‐controlled room (18–24 °C) which was subject to weekly cleaning and dusting of all surfaces. Every two weeks, the mice were transferred to new cages with fresh bedding and water bottles. Food pellets were topped up once per week.

### Mouse immunization

Soluble TmENC and E‐L2 proteins were extracted from infiltrated *N. benthamiana* plants as described previously with slight modifications [80]. Infiltrated *N. benthamiana* tissue was lyophilized and 0.3 g was extracted in 6 mL of protein extraction buffer containing only 1X PBS, 0.1% (v/v) Tween‐20, 2% (w/v) polyvinylpolypyrrolidone and 100 mm ascorbic acid. Protease inhibitors (ethylenediaminetetraccetic acid, phenylmethanesulfonylfluoride, and leupeptin) were not used in the extraction buffer as their presence could result in adverse effects in mice immunized with crude plant extract. The homogenate was sonicated in 15 s bursts for four minutes at 40% amplitude. The clarified extract was filter sterilized using a 0.2 μm syringe tip filter, flash‐frozen in liquid nitrogen, and stored at −80 °C.

Six‐ to eight‐week‐old female BALB/c mice were assigned to two treatment groups. The control group (*n* = 6) was immunized with TmENC, whereas the experimental group (*n* = 6) received E‐L2. In the experimental group, one mouse died prior to the test bleed and a second mouse died one week before the final bleed, reducing the sample size to four.

Prior to treatment, 50 μL of pre‐immune serum was collected from each mouse (day 0). For the primary immunization (day 1), 100 μL of crude plant extract containing ~ 10 μg of antigen was emulsified with 100 μL of Complete Freund's adjuvant and 50 μL was injected subcutaneously in four different sites (two injections at the front legs and two injections at the hind legs) for a total injection volume of 200 μL. Three booster doses (~ 10 μg each) were administered following the same method on days 21, 42, and 49, with the antigen emulsified with 100 μL Incomplete Freund's adjuvant. The mice were terminated on day 56 to collect the final bleeds.

### Enzyme‐linked immunosorbent assays (ELISAs) for antibody response

MaxiSorp 96‐well plates (Thermo Fisher Scientific Inc., Waltham, MA, USA) were coated with either SEC purified E‐L2 or synthetic L2 peptide at a concentration of 2 μg/mL in a 0.1 m carbonate–bicarbonate buffer (pH 9.4). The plates were incubated overnight at 4 °C. Following four washes with 1X PBS + 0.05% Tween 20, the plates were blocked with 2% Bovine Serum Albumin (BSA) in PBS (BSA‐PBS) overnight at 4 °C. The plates were washed again and incubated with serially diluted serum (diluted in 1X PBS + 1% BSA) for three hours at room temperature. Two technical replicates were performed for each sample.

After washing, the plates were incubated with goat anti‐mouse IgG Horseradish Peroxidase (HRP)‐conjugated secondary antibody (Bio‐Rad, Hercules, CA, USA, Product number: 1706516) (1 : 5000 dilution) for one hour at room temperature. After a final wash, antigen‐bound antibodies were detected using 2,2′‐azino‐bis (3‐ethylbenzothiazoline‐6‐sulfonic acid (ABTS) (Sigma‐Aldrich, Oakville, ON, Canada) and the colorimetric reaction was measured at 415 nm using an iMark™ microplate reader (Bio‐Rad, Hercules, CA, USA).

The method used by Frey *et al*. [81] was used to calculate the endpoint titer for the final bleeds of the control and experimental groups. A threshold value was calculated for each dilution using the average relative absorbance from the pre‐immune bleeds for each group, plus 2.177 (control group) or 2.631 (experimental group) times the respective standard deviation. The multiplication factors are based on a confidence level of 95% with six control samples and four experimental samples [81]. The endpoint titers were determined as the dilution where the group's average relative absorbance remained above that dilution's threshold value.

The significance of the antibody responses between the pre‐immune and final bleed of each group, and between the final bleeds of the control and experimental groups was determined using a one‐tailed Student's *t*‐test for unequal variance followed by a Holm‐Bonferroni correction for multiple comparisons. The *P‐*value of 0.05 was used to determine statistical significance.

### Construct design and cloning

The TmENC sequence was based on the entire encapsulin subunit from *T. maritima* published on Protein Data Bank (PDB: 3DKT), with the removal of the N‐terminal methionine (shown in blue in Fig. 1B). *Salmonella enterica* serovar Enteritidis (GenBank accession no. AM933172.1) was used for the identification of FepA, CirA, and IroN epitopes. Structural analysis of FepA, CirA, and IroN was performed using Phyre2 [82] and PyMOL (The PyMOL Molecular Graphics System). Candidate epitopes from FepA, CirA, and IroN were selected based on their predicted antigenicity acquired from online prediction tools VaxiJen [44], SVMTriP [45], and Antigenic Peptide Prediction from the Immunomedicine Group (http://imed.med.ucm.es/Tools/antigenic.html). Physiochemical properties of the epitopes were analyzed using DNASTAR Protean 3D (Protean 3D. Version 18.0. DNASTAR, Inc. Madison, WI).

All insertions in the TmENC subunit were flanked by flexible linkers (G_4_S) and all genes included flanking *Bsa*I sites for cloning into plant expression vectors. The L2 TmENC variants (L2‐61, L2‐124, L2‐239) were codon optimized for nuclear expression in *Nicotiana* spp. and synthesized by Bio Basic Inc. (Markham, ON, Canada). The F3, F4, C1, C2, C3, I3, and L2X3 TmENC variants were codon optimized for nuclear expression in *Nicotiana* spp. and synthesized by Twist Bioscience (South San Francisco, CA, United States).

All of the genes were cloned into modified pCaMLite‐GoldenGate‐X (pCLGG‐X) plant expression vectors [83] using Golden Gate cloning [84]. The L2 TmENC variants were cloned into chloroplast and cytosol vectors. F3 and C3 were cloned into apoplast, chloroplast, cytosol, and endoplasmic reticulum (ER) vectors. F4, C1, and C2 were cloned into the apoplast vector; I3 was cloned into the chloroplast vector; and L2X3 was cloned into the cytosol vector. All of the vectors contain a double‐enhanced *35S* promoter [85] and tobacco cryptic upstream promoter translational enhancer (*tCUP*) [86] for high expression, and the *Nos* terminator [87]. The chloroplast vector contains the chloroplast transit peptide from the small subunit of RuBisCO (RBC‐S‐TP) to target proteins to the chloroplast stroma [88]. The apoplast and ER vectors contain a PR1b signal peptide from tobacco to target proteins to the secretory pathway [89], and the ER vector contains a KDEL tetrapeptide for retrieval of the proteins to the ER [90]. The chloroplast vector contains two glycine residues after the transit peptide to stabilize the N terminus of the mature protein. The cytosol vector contains an N‐terminal methionine and two glycine residues (MGG). The cloned vectors were transformed into electrocompetent *Agrobacterium tumefaciens* EHA105 cells for transient expression in *N. benthamiana*.

### Expression of recombinant proteins

Wild‐type *N. benthamiana* plants were grown as previously described [19]. For all expression analyses, three leaves on five six‐ to eight‐week‐old *N. benthamiana* plants were agroinfiltrated via injection with a 1 : 1 mixture of *Agrobacterium* culture containing the gene of interest and a culture containing *p19*, a suppressor of post‐transcriptional gene silencing from the *Cymbidium* ringspot virus [80, 91]. Sample collection was conducted as described previously [19]. Briefly, pooled samples were collected from infiltrated plants for time‐course analyses while individual samples were collected to quantify recombinant protein accumulation, with the exception of quantification of L2X3 in which pooled samples were used.

### Analysis and quantification of recombinant protein accumulation

Total soluble proteins were isolated from infiltrated leaf tissue and analyzed via western blotting with an anti‐His monoclonal primary antibody (Takara Bio USA Inc., San Jose, CA, USA) and horseradish peroxidase‐conjugated goat anti‐mouse IgG secondary antibody (Bio‐Rad, Hercules, CA, USA, Product number: 1706516), as previously described [19].

Recombinant protein accumulation was quantified by densitometry using GelQuant software (DNR Bio‐Imaging Systems Ltd., Marienbongard, AC, Germany). The intensity of both chloroplast bands or the single apoplast or cytosol band, were compared to a dilution series consisting of known amounts of purified E‐L2, which was previously quantified against BSA on a gel stained with GelCode™ Blue Stain Reagent (Thermo Fisher Scientific, Waltham, MA, USA). The same standard was used for all blots to account for variability.

Recombinant protein accumulation levels were calculated in mg/g of leaf fresh weight (FW) by taking into consideration the mass per band calculated using GelQuant, the volume of sample loaded, the dilution factor of the protein extract, the volume of protein extraction buffer used, and the mass of leaf tissue per sample. Significance (*P‐*value *<* 0.05) was determined using a one‐tailed Student's *t*‐test for unequal variance followed by a Benjamini‐Hochberg correction for multiple comparisons.

### Purification of recombinant proteins

Recombinant proteins were purified by Immobilized Metal Affinity chromatography (IMAC) followed by size‐exclusion chromatography (SEC) as described previously [19]. Briefly, soluble plant protein extract was first passed through Nickel Sepharose 6 Fast Flow resin (GE Healthcare, Chicago, IL, USA) to capture His‐tagged recombinant proteins. The IMAC elutions containing the highest levels of recombinant protein were then further purified by size‐exclusion chromatography (SEC) using a Superose 6 Increase 10/300 GL column (GE Healthcare, Chicago, IL, USA) to isolate assembled nanoparticles and remove any remaining endogenous plant proteins.

### Transmission electron microscopy and immunogold labeling

Transmission electron microscopy and immunogold labelling was used to assess nanoparticle assembly and epitope display as described previously [19]. All grids were imaged using a JEOL JEM‐1400Flash transmission electron microscope (JEOL Canada, Ltd., Saint‐Hubert, QC, Canada).

## Author contributions

The manuscript was written through contributions of all authors. All authors have given approval to the final version of the manuscript. CAC, AK, CPG, and SS conducted protein structural analyses and designed constructs. CAC conducted all expression, purification, and assembly experiments and wrote the manuscript draft. MSD and RM conceived the project, obtained funding, and edited the manuscript.

## Conflict of interest

The authors declare no conflicts of interest.

## Data Availability

All data supporting the findings of this study are freely available upon request.

## References

[1] Kim SA , Lee Y , Ko Y , Kim S , Kim GB , Lee NK , Ahn W , Kim N , Nam GH , Lee EJ *et al*. (2023) Protein‐based nanocages for vaccine development. J Control Release 353, 767–791. 10.1016/j.jconrel.2022.12.022 36516900

[2] Nguyen B & Tolia NH (2021) Protein‐based antigen presentation platforms for nanoparticle vaccines. NPJ Vaccines 6, 70. 10.1038/s41541-021-00330-7 PMC8119681 33986287

[3] Lopez‐Sagaseta J , Malito E , Rappuoli R & Bottomley MJ (2016) Self‐assembling protein nanoparticles in the design of vaccines. Comput Struct Biotechnol J 14, 58–68. 10.1016/j.csbj.2015.11.001 PMC4706605 26862374

[4] Bhaskar S & Lim S (2017) Engineering protein nanocages as carriers for biomedical applications. NPG Asia Mater 9, e371. 10.1038/am.2016.128 PMC7091667 32218880

[5] Pandey KK , Sahoo BR & Pattnaik AK (2024) Protein nanoparticles as vaccine platforms for human and zoonotic viruses. Viruses 16, 10.3390/v16060936 PMC11209504 38932228

[6] Neek M , Kim TI & Wang SW (2019) Protein‐based nanoparticles in cancer vaccine development. Nanomedicine 15, 164–174. 10.1016/j.nano.2018.09.004 PMC6289732 30291897

[7] Kwon S & Giessen TW (2025) Engineering encapsulin nanocages for drug delivery. Mater Adv 6, 6209–6220. 10.1039/d5ma00386e PMC12285755 40717769

[8] Giessen TW (2016) Encapsulins: microbial nanocompartments with applications in biomedicine, nanobiotechnology and materials science. Curr Opin Chem Biol 34, 1–10. 10.1016/j.cbpa.2016.05.013 27232770

[9] Rennie C , Sives C , Boyton I , Diaz D , Gorrie C , Vittorio O , Collins‐Praino L & Care A (2024) In vivo behavior of systemically administered Encapsulin protein Nanocages and implications for their use in targeted drug delivery. Advanced Therapeutics 7, 2300360.

[10] Cassidy‐Amstutz C , Oltrogge L , Going CC , Lee A , Teng P , Quintanilla D , East‐Seletsky A , Williams ER & Savage DF (2016) Identification of a minimal peptide tag for in vivo and in vitro loading of Encapsulin. Biochemistry 55, 3461–3468. 10.1021/acs.biochem.6b00294 27224728

[11] Gabashvili AN , Chmelyuk NS , Efremova MV , Malinovskaya JA , Semkina AS & Abakumov MA (2020) Encapsulins‐bacterial protein Nanocompartments: structure, properties, and application. Biomolecules 10, 60966. 10.3390/biom10060966 PMC7355545 32604934

[12] Lagoutte P , Mignon C , Stadthagen G , Potisopon S , Donnat S , Mast J , Lugari A & Werle B (2018) Simultaneous surface display and cargo loading of encapsulin nanocompartments and their use for rational vaccine design. Vaccine 36, 3622–3628. 10.1016/j.vaccine.2018.05.034 29759379

[13] Choi H , Eom S , Kim HU , Bae Y , Jung HS & Kang S (2021) Load and display: engineering Encapsulin as a modular Nanoplatform for protein‐cargo encapsulation and protein‐ligand decoration using Split Intein and SpyTag/SpyCatcher. Biomacromolecules 22, 3028–3039. 10.1021/acs.biomac.1c00481 34142815

[14] Moon H , Lee J , Min J & Kang S (2014) Developing genetically engineered encapsulin protein cage nanoparticles as a targeted delivery nanoplatform. Biomacromolecules 15, 3794–3801. 10.1021/bm501066m 25180761

[15] Sutter M , Boehringer D , Gutmann S , Gunther S , Prangishvili D , Loessner MJ , Stetter KO , Weber‐Ban E & Ban N (2008) Structural basis of enzyme encapsulation into a bacterial nanocompartment. Nat Struct Mol Biol 15, 939–947. 10.1038/nsmb.1473 19172747

[16] Choi B , Moon H , Hong SJ , Shin C , Do Y , Ryu S & Kang S (2016) Effective delivery of antigen‐Encapsulin nanoparticle fusions to dendritic cells leads to antigen‐specific cytotoxic T cell activation and tumor rejection. ACS Nano 10, 7339–7350. 10.1021/acsnano.5b08084 27390910

[17] Bae Y , Kim GJ , Kim H , Park SG , Jung HS & Kang S (2018) Engineering tunable dual functional protein cage nanoparticles using bacterial superglue. Biomacromolecules 19, 2896–2904. 10.1021/acs.biomac.8b00457 29847113

[18] Lee TH , Carpenter TS , D'Haeseleer P , Savage DF & Yung MC (2020) Encapsulin carrier proteins for enhanced expression of antimicrobial peptides. Biotechnol Bioeng 117, 603–613. 10.1002/bit.27222 31709513

[19] Charron CA , Kaldis A , Shamriz S , Renaud JB , Diarra MS , Garnham CP & Menassa R (2026) Plant‐produced encapsulin displays non‐typhoidal salmonella enterica antigens and assembles into mosaic nanoparticles. FEBS J 293, 1908–1929. 10.1111/febs.70340 PMC13044985 41264285

[20] Michel‐Souzy S , Hamelmann NM , Zarzuela‐Pura S , Paulusse JMJ & Cornelissen J (2021) Introduction of surface loops as a tool for Encapsulin functionalization. Biomacromolecules 22, 5234–5242. 10.1021/acs.biomac.1c01156 PMC8672354 34747611

[21] Buchanan SK , Smith BS , Venkatramani L , Xia D , Esser L , Palnitkar M , Chakraborty R , van der Helm D & Deisenhofer J (1999) Crystal structure of the outer membrane active transporter FepA from Escherichia coli. Nat Struct Biol 6, 56–63. 10.1038/4931 9886293

[22] Clarke TE , Tari LW & Vogel HJ (2001) Structural biology of bacterial iron uptake systems. Curr Top Med Chem 1, 7–30. 10.2174/1568026013395623 11895294

[23] Lin J , Hogan JS & Smith KL (1998) Inhibition of in vitro growth of coliform bacteria by a monoclonal antibody directed against ferric enterobactin receptor FepA. J Dairy Sci 81, 1267–1274. 10.3168/jds.S0022-0302(98)75688-7 9621228

[24] Frank SA (2002) Specificity and Cross‐Reactivity. In Immunology and Evolution of Infectious Disease Princeton University Press, Princeton (NJ). 20821852

[25] Tada T , Peng JY , Dcosta BM & Landau NR (2023) Single‐epitope T cell‐based vaccine protects against SARS‐CoV‐2 infection in a preclinical animal model. JCI Insight 8, 167306. 10.1172/jci.insight.167306 PMC10132166 37036004

[26] Testa JS & Philip R (2012) Role of T‐cell epitope‐based vaccine in prophylactic and therapeutic applications. Future Virol 7, 1077–1088. 10.2217/fvl.12.108 PMC3636528 23630544

[27] Chekabab SM , Rehman MA , Yin X , Carrillo C , Mondor M & Diarra MS (2019) Growth of salmonella enterica Serovars typhimurium and Enteritidis in iron‐poor media and in meat: role of Catecholate and Hydroxamate Siderophore transporters. J Food Prot 82, 548–560. 10.4315/0362-028X.JFP-18-371 30901525

[28] Snijder J , Kononova O , Barbu IM , Uetrecht C , Rurup WF , Burnley RJ , Koay MS , Cornelissen JJ , Roos WH , Barsegov V *et al*. (2016) Assembly and mechanical properties of the cargo‐free and cargo‐loaded bacterial Nanocompartment Encapsulin. Biomacromolecules 17, 2522–2529. 10.1021/acs.biomac.6b00469 27355101

[29] Jung HG , Jeong S , Kang MJ , Hong I , Park YS , Ko E , Kim JO & Choi DY (2024) Molecular Design of Encapsulin Protein Nanoparticles to display rotavirus antigens for enhancing immunogenicity. Vaccines (Basel) 12, 91020. 10.3390/vaccines12091020 PMC11435836 39340050

[30] Rybicki EP (2014) Plant‐based vaccines against viruses. Virol J 11, 205. 10.1186/s12985-014-0205-0 PMC4264547 25465382

[31] Chen Q & Davis KR (2016) The potential of plants as a system for the development and production of human biologics. F1000Res 5, F1000. 10.12688/f1000research.8010.1 PMC4876878 27274814

[32] Tschofen M , Knopp D , Hood E & Stoger E (2016) Plant molecular farming: much more than medicines. Annu Rev Anal Chem (Palo Alto, Calif) 9, 271–294. 10.1146/annurev-anchem-071015-041706 27049632

[33] Stoger E , Fischer R , Moloney M & Ma JK (2014) Plant molecular pharming for the treatment of chronic and infectious diseases. Annu Rev Plant Biol 65, 743–768. 10.1146/annurev-arplant-050213-035850 24579993

[34] Marsian J & Lomonossoff GP (2016) Molecular pharming ‐ VLPs made in plants. Curr Opin Biotechnol 37, 201–206. 10.1016/j.copbio.2015.12.007 26773389

[35] Balke I & Zeltins A (2020) Recent advances in the use of plant virus‐like particles as vaccines. Viruses 12, 10.3390/v12030270 PMC7150928 32121192

[36] Strasser R (2016) Plant protein glycosylation. Glycobiology 26, 926–939. 10.1093/glycob/cww023 PMC5045529 26911286

[37] Margolin E , Verbeek M , de Moor W , Chapman R , Meyers A , Schafer G , Williamson AL & Rybicki E (2021) Investigating constraints along the plant secretory pathway to improve production of a SARS‐CoV‐2 spike vaccine candidate. Front Plant Sci 12, 798822. 10.3389/fpls.2021.798822 PMC8764404 35058959

[38] Kolotilin I , Topp E , Cox E , Devriendt B , Conrad U , Joensuu J , Stoger E , Warzecha H , McAllister T , Potter A *et al*. (2014) Plant‐based solutions for veterinary immunotherapeutics and prophylactics. Vet Res 45, 117. 10.1186/s13567-014-0117-4 PMC4280687 25559098

[39] Topp E , Irwin R , McAllister T , Lessard M , Joensuu JJ , Kolotilin I , Conrad U , Stoger E , Mor T , Warzecha H *et al*. (2016) The case for plant‐made veterinary immunotherapeutics. Biotechnol Adv 34, 597–604. 10.1016/j.biotechadv.2016.02.007 26875776

[40] Newton SM , Igo JD , Scott DC & Klebba PE (1999) Effect of loop deletions on the binding and transport of ferric enterobactin by FepA. Mol Microbiol 32, 1153–1165. 10.1046/j.1365-2958.1999.01424.x 10383757

[41] Phan HT , Pham VT , Ho TT , Pham NB , Chu HH , Vu TH , Abdelwhab EM , Scheibner D , Mettenleiter TC , Hanh TX *et al*. (2020) Immunization with plant‐derived multimeric H5 hemagglutinins protect chicken against highly pathogenic avian influenza virus H5N1. Vaccines (Basel) 8, 8040593. 10.3390/vaccines8040593 PMC7712794 33050224

[42] Rath A , Glibowicka M , Nadeau VG , Chen G & Deber CM (2009) Detergent binding explains anomalous SDS‐PAGE migration of membrane proteins. Proc Natl Acad Sci USA 106, 1760–1765. 10.1073/pnas.0813167106 PMC2644111 19181854

[43] Giessen TW (2024) The structural diversity of Encapsulin protein shells. Chembiochem 25, e202400535. 10.1002/cbic.202400535 PMC11664910 39330624

[44] Doytchinova IA & Flower DR (2007) VaxiJen: a server for prediction of protective antigens, tumour antigens and subunit vaccines. BMC Bioinformatics 8, 4. 10.1186/1471-2105-8-4 PMC1780059 17207271

[45] Yao B , Zhang L , Liang S & Zhang C (2012) SVMTriP: a method to predict antigenic epitopes using support vector machine to integrate tri‐peptide similarity and propensity. PLoS One 7, e45152. 10.1371/journal.pone.0045152 PMC3440317 22984622

[46] Nooraei S , Bahrulolum H , Hoseini ZS , Katalani C , Hajizade A , Easton AJ & Ahmadian G (2021) Virus‐like particles: preparation, immunogenicity and their roles as nanovaccines and drug nanocarriers. J Nanobiotechnology 19, 59. 10.1186/s12951-021-00806-7 PMC7905985 33632278

[47] Van Tilbeurgh M , Lemdani K , Beignon AS , Chapon C , Tchitchek N , Cheraitia L , Marcos Lopez E , Pascal Q , Le Grand R , Maisonnasse P *et al*. (2021) Predictive markers of immunogenicity and efficacy for human vaccines. Vaccines (Basel) 9, 10.3390/vaccines9060579 PMC8226531 34205932

[48] Alfano EF , Lentz EM , Bellido D , Dus Santos MJ , Goldbaum FA , Wigdorovitz A & Bravo‐Almonacid FF (2015) Expression of the multimeric and highly immunogenic Brucella spp. Lumazine synthase fused to bovine rotavirus VP8d as a scaffold for antigen production in tobacco chloroplasts. Front Plant Sci 6, 1170. 10.3389/fpls.2015.01170 PMC4688359 26779198

[49] Liang J , Yao L , Liu Z , Chen Y , Lin Y & Tian T (2025) Nanoparticles in subunit vaccines: immunological foundations, categories, and applications. Small 21, e2407649. 10.1002/smll.202407649 39501996

[50] Jung W & Son YM (2025) Nanoparticle‐driven modulation of mucosal immunity and interplay with the microbiome. J Microbiol Biotechnol 35, e2404033. 10.4014/jmb.2504.04033 PMC12197821 40537897

[51] Cappelli L , Cinelli P , Giusti F , Ferlenghi I , Utrio‐Lanfaloni S , Wahome N , Bottomley MJ , Maione D & Cozzi R (2022) Self‐assembling protein nanoparticles and virus like particles correctly display beta‐barrel from meningococcal factor H‐binding protein through genetic fusion. PLoS One 17, e0273322. 10.1371/journal.pone.0273322 PMC9480994 36112575

[52] Zarate‐Bonilla LJ , Del Portillo P , Saenz‐Suarez H , Gonzales‐Santos J , Barreto‐Sampaio GE , Poutou‐Pinales RA , Rey AF & Rey JG (2014) Computational modeling and preliminary iroN, fepA, and cirA gene expression in salmonella Enteritidis under iron‐deficiency‐induced conditions. Poult Sci 93, 221–230. 10.3382/ps.2012-02993 24570443

[53] Larrie‐Bagha SM , Rasooli I , Mousavi‐Gargari SL , Rasooli Z & Nazarian S (2013) Passive immunization by recombinant ferric enterobactin protein (FepA) from Escherichia coli O157. Iran J Microbiol 5, 113–119. PMC3696845 23825727

[54] Baghal SM , Gargari SL & Rasooli I (2010) Production and immunogenicity of recombinant ferric enterobactin protein (FepA). Int J Infect Dis 14 (Suppl 3), e166–e170. 10.1016/j.ijid.2009.12.009 20418143

[55] Sood S , Rishi P , Vohra H , Sharma S & Ganguly NK (2005) Cellular immune response induced by salmonella enterica serotype Typhi iron‐regulated outer‐membrane proteins at peripheral and mucosal levels. J Med Microbiol 54, 815–821. 10.1099/jmm.0.46042-0 16091431

[56] Williams PH , Rabsch W , Methner U , Voigt W , Tschape H & Reissbrodt R (2006) Catecholate receptor proteins in salmonella enterica: role in virulence and implications for vaccine development. Vaccine 24, 3840–3844. 10.1016/j.vaccine.2005.07.020 16154248

[57] Kaneshige T , Yaguchi K & Ohgitani T (2009) Siderophore receptor IroN is an important protective antigen against salmonella infection in chickens. Avian Dis 53, 563–567. 10.1637/8925-051309-Reg.1 20095157

[58] Zhang Y , Ru Y , Zhao L , Hao R , Yang Y , Li Y , Zhang R , Jiang C & Zheng H (2025) Ferritin and Encapsulin nanoparticles as effective vaccine delivery systems: boosting the immunogenicity of the African swine fever virus C129R protein. Viruses 17, 17040556. 10.3390/v17040556 PMC12031609 40284999

[59] Xiong X , Sun C , Vago FS , Klose T , Zhu J & Jiang W (2020) Cryo‐EM structure of heterologous protein complex loaded Thermotoga Maritima Encapsulin capsid. Biomolecules 10, 10091342. 10.3390/biom10091342 PMC7565527 32961724

[60] Giessen TW (2022) Encapsulins. Annu Rev Biochem 91, 353–380. 10.1146/annurev-biochem-040320-102858 PMC9944552 35303791

[61] Scott NR , Mann B , Tuomanen EI & Orihuela CJ (2021) Multi‐valent protein hybrid pneumococcal vaccines: a strategy for the next generation of vaccines. Vaccines (Basel) 9, 9030209. 10.3390/vaccines9030209 PMC8002124 33801372

[62] Rella SA , Kulikova YA , Minnegalieva AR & Kondrashov FA (2024) Complex vaccination strategies prevent the emergence of vaccine resistance. Evolution 78, 1722–1738. 10.1093/evolut/qpae106 38990788

[63] Moyle PM & Toth I (2013) Modern subunit vaccines: development, components, and research opportunities. ChemMedChem 8, 360–376. 10.1002/cmdc.201200487 23316023

[64] Correia BE , Bates JT , Loomis RJ , Baneyx G , Carrico C , Jardine JG , Rupert P , Correnti C , Kalyuzhniy O , Vittal V *et al*. (2014) Proof of principle for epitope‐focused vaccine design. Nature 507, 201–206. 10.1038/nature12966 PMC4260937 24499818

[65] Bhatnager R , Bhasin M , Arora J & Dang AS (2021) Epitope based peptide vaccine against SARS‐COV2: an immune‐informatics approach. J Biomol Struct Dyn 39, 5690–5705. 10.1080/07391102.2020.1787227 32619134

[66] Halfmann PJ , Patel RS , Loeffler K , Yasuhara A , Van De Velde LA , Yang JE , Chervin J , Troxell C , Huang M , Zheng N *et al*. (2025) Multivalent S2 subunit vaccines provide broad protection against Clade 1 sarbecoviruses in female mice. Nat Commun 16, 462. 10.1038/s41467-025-55824-y PMC11706982 39774966

[67] Yang X , Sun Y , Gu H , Li D , Zhang L , Li T & Wang H (2024) Multi‐component protein vaccine induces a Strong and long‐term immune response against Monkeypox virus. Vaccines (Basel) 12, 12121410. 10.3390/vaccines12121410 PMC11680392 39772071

[68] de Araujo LP , Silva EN , de Alencar SM , Corsetti PP & de Almeida LA (2025) Multivalent vaccine candidate from conserved immunogenic peptides in entry or exit proteins of Orthopoxvirus genus. Sci Rep 15, 12503. 10.1038/s41598-025-96755-4 PMC11992193 40216856

[69] Mosa AI , Campo DS , Khudyakov Y , AbouHaidar MG , Gehring AJ , Zahoor A , Ball JK , Urbanowicz RA & Feld JJ (2023) Polyvalent immunization elicits a synergistic broadly neutralizing immune response to hypervariable region 1 variants of hepatitis C virus. Proc Natl Acad Sci USA 120, e2220294120. 10.1073/pnas.2220294120 PMC10268328 37276424

[70] Krammer F (2020) SARS‐CoV‐2 vaccines in development. Nature 586, 516–527. 10.1038/s41586-020-2798-3 32967006

[71] Ripka JF , Perez‐Riba A , Chaturbedy PK & Itzhaki LS (2021) Testing the length limit of loop grafting in a helical repeat protein. Curr Res Struct Biol 3, 30–40. 10.1016/j.crstbi.2020.12.002 PMC8244534 34235484

[72] Vasyagin EA , Mardanova ES & Ravin NV (2025) Virus‐like particles formed by the coat protein of the single‐stranded RNA phage PQ465 as a carrier for antigen presentation. Molecules 30, 10.3390/molecules30204056 PMC12566596 41157072

[73] Caldeira JC & Peabody DS (2011) Thermal stability of RNA phage virus‐like particles displaying foreign peptides. J Nanobiotechnology 9, 22. 10.1186/1477-3155-9-22 PMC3118325 21609437

[74] Zhou HX & Pang X (2018) Electrostatic interactions in protein structure, folding, binding, and condensation. Chem Rev 118, 1691–1741. 10.1021/acs.chemrev.7b00305 PMC5831536 29319301

[75] Chan P , Curtis RA & Warwicker J (2013) Soluble expression of proteins correlates with a lack of positively‐charged surface. Sci Rep 3, 3333. 10.1038/srep03333 PMC6506438 24276756

[76] Gostaviceanu A , Gavrilas S , Copolovici L & Copolovici DM (2023) Membrane‐active peptides and their potential biomedical application. Pharmaceutics 15, 2091. 10.3390/pharmaceutics15082091 PMC10459175 37631305

[77] Raghunathan G , Sokalingam S , Soundrarajan N , Madan B , Munussami G & Lee SG (2013) Modulation of protein stability and aggregation properties by surface charge engineering. Mol BioSyst 9, 2379–2389. 10.1039/c3mb70068b 23861008

[78] Luo H , Ma Y , Bi J , Li Z , Wang Y , Su Z , Gerstweiler L , Ren Y & Zhang S (2024) Experimental and molecular dynamics simulation studies on the physical properties of three HBc‐VLP derivatives as nanoparticle protein vaccine candidates. Vaccine 42, 125992. 10.1016/j.vaccine.2024.05.040 38811268

[79] McCraw DM , Myers ML , Gulati NM , Prabhakaran M , Brand J , Andrews S , Gallagher JR , Maldonado‐Puga S , Kim AJ , Torian U *et al*. (2023) Designed nanoparticles elicit cross‐reactive antibody responses to conserved influenza virus hemagglutinin stem epitopes. PLoS Pathog 19, e1011514. 10.1371/journal.ppat.1011514 PMC10491405 37639457

[80] Miletic S , Simpson DJ , Szymanski CM , Deyholos MK & Menassa R (2015) A plant‐produced bacteriophage Tailspike protein for the control of salmonella. Front Plant Sci 6, 1221. 10.3389/fpls.2015.01221 PMC4705272 26779243

[81] Frey A , Di Canzio J & Zurakowski D (1998) A statistically defined endpoint titer determination method for immunoassays. J Immunol Methods 221, 35–41. 10.1016/s0022-1759(98)00170-7 9894896

[82] Kelley LA , Mezulis S , Yates CM , Wass MN & Sternberg MJ (2015) The Phyre2 web portal for protein modeling, prediction and analysis. Nat Protoc 10, 845–858. 10.1038/nprot.2015.053 PMC5298202 25950237

[83] VanderBurgt JT , Harper O , Garnham CP , Kohalmi SE & Menassa R (2023) Plant production of a virus‐like particle‐based vaccine candidate against porcine reproductive and respiratory syndrome. Front Plant Sci 14, 1044675. 10.3389/fpls.2023.1044675 PMC9902946 36760639

[84] Marillonnet S & Werner S (2015) Assembly of multigene constructs using Golden Gate cloning. Methods Mol Biol 1321, 269–284. 10.1007/978-1-4939-2760-9_19 26082229

[85] Kay R , Chan A , Daly M & McPherson J (1987) Duplication of CaMV 35S promoter sequences creates a strong enhancer for plant genes. Science 236, 1299–1302. 10.1126/science.236.4806.1299 17770331

[86] Wu K , Malik K , Tian L , Hu M , Martin T , Foster E , Brown D & Miki B (2001) Enhancers and core promoter elements are essential for the activity of a cryptic gene activation sequence from tobacco, tCUP. Mol Gen Genomics 265, 763–770. 10.1007/s004380100478 11523793

[87] Bevan M , Barnes WM & Chilton MD (1983) Structure and transcription of the nopaline synthase gene region of T‐DNA. Nucleic Acids Res 11, 369–385. 10.1093/nar/11.2.369 PMC325720 6298724

[88] Menassa R , Kennette W , Nguyen V , Rymerson R , Jevnikar A & Brandle J (2004) Subcellular targeting of human interleukin‐10 in plants. J Biotechnol 108, 179–183. 10.1016/j.jbiotec.2003.11.009 15129727

[89] Cutt JR , Dixon DC , Carr JP & Klessig DF (1988) Isolation and nucleotide sequence of cDNA clones for the pathogenesis‐related proteins PR1a, PR1b and PR1c of Nicotiana tabacum cv. Xanthi nc induced by TMV infection. Nucleic Acids Res 16, 9861. 10.1093/nar/16.20.9861 PMC338789 3186451

[90] Pelham HR (1990) The retention signal for soluble proteins of the endoplasmic reticulum. Trends Biochem Sci 15, 483–486. 10.1016/0968-0004(90)90303-s 2077689

[91] Silhavy D , Molnar A , Lucioli A , Szittya G , Hornyik C , Tavazza M & Burgyan J (2002) A viral protein suppresses RNA silencing and binds silencing‐generated, 21‐ to 25‐nucleotide double‐stranded RNAs. EMBO J 21, 3070–3080. 10.1093/emboj/cdf312 PMC125389 12065420

